# Biochemical and Multi-Omics Approaches To Obtain Molecular Insights into the Catabolism of the Plasticizer Benzyl Butyl Phthalate in *Rhodococcus* sp. Strain PAE-6

**DOI:** 10.1128/spectrum.04801-22

**Published:** 2023-06-15

**Authors:** Suman Basu, Rinita Dhar, Mousumi Bhattacharyya, Tapan K. Dutta

**Affiliations:** a Department of Microbiology, Bose Institute, Kolkata, West Bengal, India; Chung-Ang University

**Keywords:** phthalic acid esters, benzyl butyl phthalate, *Rhodococcus*, biodegradation, hydrolase, multi-omics analysis

## Abstract

Phthalate diesters are extensively used as plasticizers in manufacturing plastic materials; however, because of their estrogenic properties, these chemicals have emerged as a global threat to human health. The present study investigated the course of degradation of a widely used plasticizer, benzyl butyl phthalate (BBP), by the bacterium PAE-6, belonging to the genus *Rhodococcus*. The metabolism of BBP, possessing structurally dissimilar side chains, was evaluated biochemically using a combination of respirometric, chromatographic, enzymatic, and mass-spectrometric analyses, depicting pathways of degradation. Consequently, the biochemical observations were corroborated by identifying possible catabolic genes from whole-genome analysis, and the involvement of inducible specific esterases and other degradative enzymes was validated by transcriptomic, reverse transcription-quantitative PCR (RT-qPCR) and proteomic analyses. Nonetheless, phthalic acid (PA), an intermediate of BBP, could not be efficiently metabolized by strain PAE-6, although the genome contains a PA-degrading gene cluster. This deficiency of complete degradation of BBP by strain PAE-6 was effectively managed by using a coculture of strains PAE-6 and PAE-2. The latter was identified as a *Paenarthrobacter* strain which can efficiently utilize PA. Based on sequence analysis of the PA-degrading gene cluster in strain PAE-6, it appeared that the alpha subunit of the multicomponent phthalate 3,4-dioxygenase harbors a number of altered residues in the multiple sequence alignment of homologous subunits, which may play a role(s) in poor turnover of PA.

**IMPORTANCE** Benzyl butyl phthalate (BBP), an estrogenic, high-molecular-weight phthalic acid diester, is an extensively used plasticizer throughout the world. Due to its structural rigidity and hydrophobic nature, BBP gets adsorbed on sediments and largely escapes the biotic and abiotic degradative processes of the ecosystem. In the present study, a potent BBP-degrading bacterial strain belonging to the genus *Rhodococcus* was isolated that can also assimilate a number of other phthalate diesters of environmental concern. Various biochemical and multi-omics analyses revealed that the strain harbors all the required catabolic machinery for the degradation of the plasticizer and elucidated the inducible regulation of the associated catabolic genes and gene clusters.

## INTRODUCTION

In the modern lifestyle, there has been an inevitable increase in the use of plastics worldwide. This has turned out to be one of the more catastrophic situations for our surrounding environments, with a massive threat to all forms of life, including humans. Plasticizers are the preferred additives in the manufacture of various plastic materials, such as polyvinyl chloride, polycarbonate, epoxy resins, and other polymeric materials. Plasticizers are used to impart softness, flexibility, shine, and durability to polymeric products. Plasticizers are high-production-volume synthesized chemicals, and phthalic acid esters (PAEs) are one of the major groups of chemicals used worldwide since the 1920s (1, 2). Due to their massive and widespread usage in many daily usable products, *viz.*, plastic packaging materials (bags, bottles, and containers), toys, paints, cosmetics, food cans, pesticides, detergents, etc., PAEs are ubiquitous in the environment. Since these toxic PAEs are noncovalently bound to the plastic or polymer matrices, they can easily leach out into the surrounding environment, eventually posing a threat to the well-being of humans, aquatic life, and terrestrial life forms (3). Specifically, through interference with the estrogenic system by hormone mimicry, PAEs can disrupt the endocrine system, causing several health problems, including the development of reproductive toxicity, neurodevelopmental disorders, and even cancer (3).

Even though phthalates are present below the toxic limit in some environments, quite often, they are bioaccumulated via the food chain and eventually are biomagnified to a much higher level. The concentration of hydrophobic PAEs far exceeds their water solubility (4, 5). The U.S. Environmental Protection Agency (EPA) and the European Union (EU) have listed six PAEs as priority pollutants, which include dimethyl phthalate (DMP), diethyl phthalate (DEP), di-*n*-butyl phthalate (DBP), benzyl butyl phthalate (BBP), di-*n*-octyl phthalate (DOP), and di-(2-ethylhexyl) phthalate (DEHP) (6, 7). Among them, BBP was identified as a priority environmental pollutant and reported as hazardous based on its toxicity for aquatic and terrestrial organisms (3). In addition to BBP’s significant reproductive and developmental toxicity, the EPA has classified BBP as a priority pollutant and a probable and possible cancer-causing agent in humans (8, 9). Nevertheless, no experimental data in the atmospheric photodegradation of BBP are available in the context of abiotic processes. However, a study of photodegradation of BBP in an aqueous solution reported less than 5% degradation in 28 days (10). Thus, zero discharge and the removal of these toxic synthetic contaminants have turned out to be major global concerns in protecting life and the environment.

Microbial degradation being the most effective and ecofriendly approach, during the past few decades, metabolic pathways, kinetics, and related catabolic mechanisms in the degradation of several phthalate esters have been investigated in a broad range of bacterial species (3, 11). Nevertheless, most of the studies focused on the biodegradation of low-molecular-weight dialkyl phthalates. On the other hand, relatively more hydrophobic and persistent high-molecular-weight dialkyl phthalates and alkyl aryl phthalates such as BBP are poorly studied at the biochemical and molecular levels.

In the present study, the biochemical characterization of metabolic pathways for the degradation of BBP in *Rhodococcus* sp. strain PAE-6 was carried out with the corroboration of data obtained from multi-omics analyses, thereby sorting out the participating inducible catabolic genes and enzymes. In addition, the poor ability of strain PAE-6 to metabolize PA was shown to be due to the aberration of amino acid residues at the sequence level of the catalytic subunit of phthalate dioxygenase (PDO), and the shortfall was compensated for by using an efficient PA-utilizing strain belonging to the genus *Paenarthrobacter* in a coculture, resulting in the complete assimilation of BBP.

## RESULTS

### Isolation and characterization of a BBP-degrading strain.

**FIG 1:**
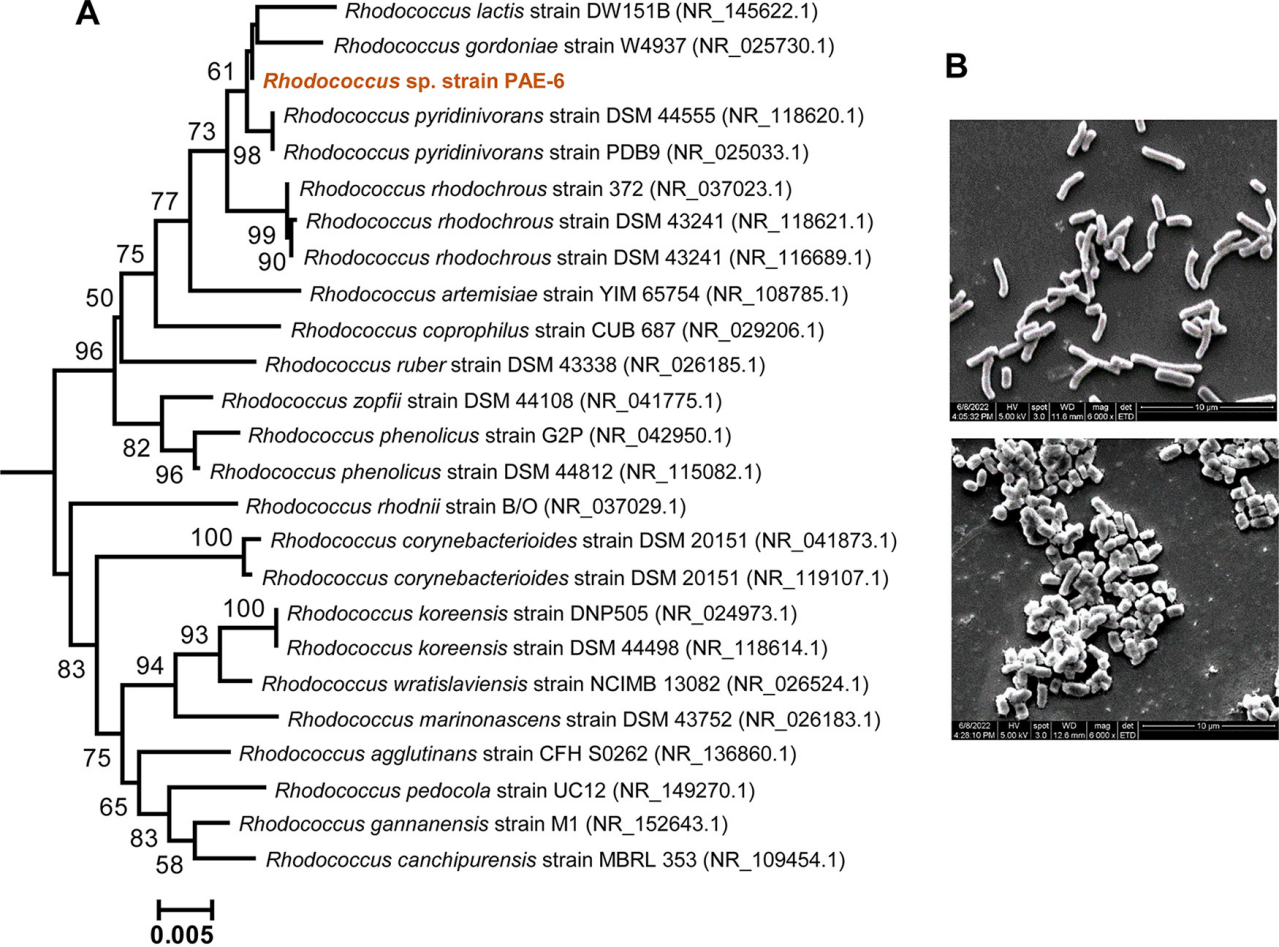
(A) Phylogenetic relationship based on 16S rRNA gene sequences of PAE-6 and representative strains of the genus *Rhodococcus*. Numbers at the nodes indicate the levels of bootstrap support based on neighbor joining analysis of 100 resampled data sets. Bootstrap values below 50% are not shown. The scale bar represents 0.005 substitution per nucleotide position. GenBank accession numbers of the sequences are given in parentheses. The multiple-sequence alignment was performed using ClustalX2, and the phylogenetic tree was constructed using a neighbor joining algorithm as implemented in Tree Explorer 2.12. Strain PAE-6 is indicated in red boldface type. (B) Scanning electron-microscopic images of log-phase cells of strain PAE-6 grown in the presence of succinate (top) and BBP (bottom). Magnification, ×6,000.

Enrichment of a municipal-waste-contaminated soil sample from Kolkata in the presence of BBP led to the isolation of bacterial strains designated PAE-6 and PAE-2. The amplified 16S rRNA sequence of strain PAE-6 and that from whole-genome sequence data revealed 99.93% sequence identity with Rhodococcus pyridinivorans strain DSM 44555 (NR118620.1), a pyridine utilizing strain (12). Average nucleotide identity (ANI) analysis revealed that strain PAE-6 is phylogenetically related to Rhodococcus pyridinivorans NBRC 100608 with 98.57% identity at the genome level. However, strain PAE-6 could not utilize pyridine. On the other hand, 100% sequence identity to other housekeeping genes, such as *gyrB*, *rpoD*, *dnaK*, *trpB*, and *recA*, was also found. A phylogenetic tree of strain PAE-6 with the closest neighbors in terms of 16S rRNA gene sequence similarity is shown in Fig. 1A. Based on the above results, the test organism was designated *Rhodococcus* sp. strain PAE-6. The morphological features of strain PAE-6, as determined by scanning electron microscopy under different nutrient conditions, are depicted in Fig. 1B. Succinate-grown cells of strain PAE-6 appeared rod-shaped with a smooth surface and were well dispersed. In contrast, the strain grown in the presence of BBP showed cells that were smaller and appeared as clumps, formed due to autoaggregation. These differential morphological patterns may be due to stresses imposed by BBP, a hydrophobic aromatic compound (13).

### Substrate utilization potential of strain PAE-6.

**FIG 2:**
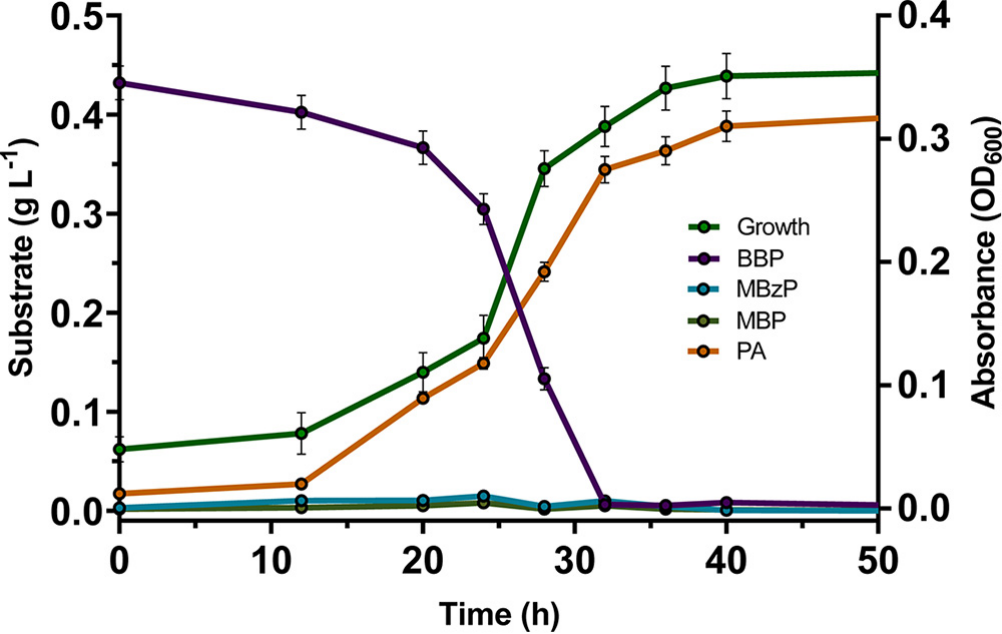
Growth curve of *Rhodococcus* sp. strain PAE-6 upon utilization of BBP as the sole carbon source under optimal growth conditions. The accumulation of metabolites is indicated with different-colored lines. Vertical bars represent standard deviations (SD) of mean values from triplicate measurements.

*Rhodococcus* sp. strain PAE-6 was found to utilize BBP as a sole source of carbon and energy, and the optimum temperature for growth in mineral salt medium (MSM; pH 7.0) was 28°C with 0.5 g L^−1^ of BBP under shaking culture conditions (data not shown). Other than BBP, strain PAE-6 can utilize several phthalate diesters, namely, DMP, DEP, DBP, DEHP, and DOP. Table 1 shows the catabolic potential of strain PAE-6 for various PAEs and the possible metabolic intermediates of BBP based on the growth of the strain at the expense of the PAEs, used individually as sole sources of carbon and energy. The utilization of BBP was confirmed by its elimination from MSM-BBP spent culture supernatant, with the concomitant increase in bacterial biomass (Fig. 2). BBP was found to be completely utilized within 36 h of incubation, and the growth rate of strain PAE-6 was found to be 0.04 h^−1^ during the log phase of its growth under optimal conditions. Despite the ability of strain PAE-6 to utilize various structurally distinct dialkyl- and alkylaryl phthalate diesters and BBP-hydrolyzed side chain alcohols, strain PAE-6 showed inadequate utilization of PA as a sole carbon source. It has been observed that there was an accumulation of PA during growth in the presence of BBP. On the other hand, no significant accumulation of mono-*n*-butyl phthalate (MBP) or monobenzyl phthalate (MBzP) was observed during the growth on BBP. Thus, the accumulation of phthalic acid (PA) in the culture medium indicated that the latter emerged as a poorly degradable metabolite in the degradation of BBP by strain PAE-6 (Fig. 2).

**TABLE 1:** Substrate utilization profile for *Rhodococcus* sp. strain PAE-6^*a*^

Growth substrate	Growth rate (h^−1^)
DMP	0.17
DEP	0.17
DBP	0.17
DEHP	0.02
DOP	0.04
BBP	0.04
MBP	0.06
MBzP	0.05
PA	ND
PCA	0.08
1-Butanol	0.23
Benzyl alcohol	0.34
Benzoic acid	0.23
Butyric acid	0.23
Catechol	0.12

a The concentration of each substrate was 0.5 g L^−1^. ND, Not determined.

### Identification of BBP degradation metabolites.

**FIG 3:**
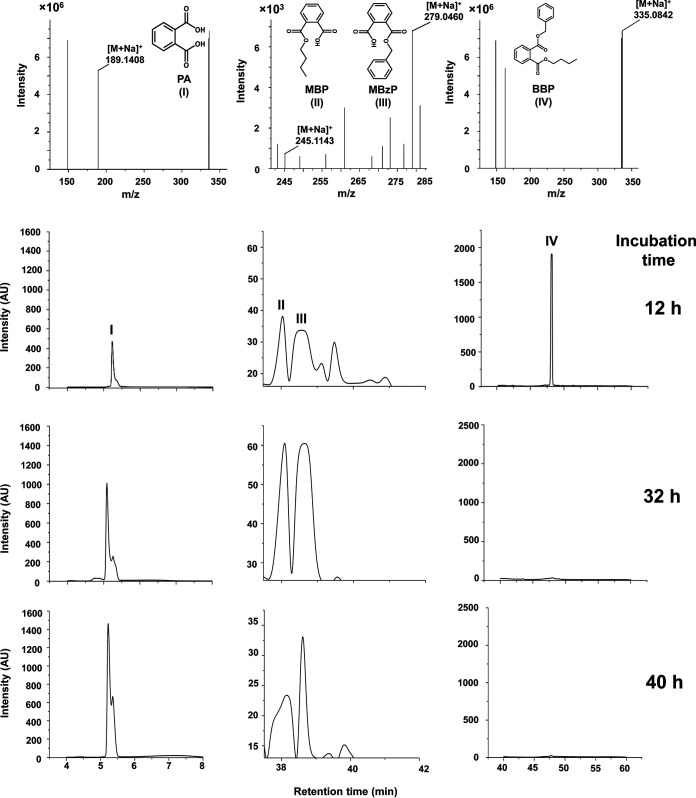
HPLC-ESI-HRMS analysis of organic extracts of spent culture of strain PAE-6 grown in the presence of BBP, showing its degradation and concomitant production of various metabolites, namely, MBP, MBzP, and PA, with incubation time.

The organic extracts of the MSM-BBP spent cultures were resolved by high-performance liquid chromatography (HPLC) to identify the BBP degradation metabolites (Fig. 3). The chromatogram indicated the presence of four distinct peaks. Based on retention time (RT), UV-visible spectra, and coelution profiles with reference compounds, peaks I to IV were identified as PA (peak I, RT = 5.2 min), MBP (peak II, RT = 38.2 min), MBzP (peak III, RT = 38.7 min), and BBP (peak IV, RT = 47.6 min), respectively. All these metabolites and the unconverted substrate were also detected in thin-layer chromatography (TLC) analysis from the same organic extract (data not shown). However, benzyl alcohol, one of the hydrolyzed products of BBP or its metabolite, could not be detected, possibly because of its transient nature in the degradation process. Likewise, the other BBP hydrolysis product, butanol, could not be seen under the conditions used. To validate the metabolic sketch, the organic extract of BBP-grown spent culture was further analyzed by direct infusion-electrospray ionization-high resolution mass spectrometry (DI-ESI-HRMS). The mass data of the analytes were correlated with the compounds identified by HPLC (Fig. 3, top). Apart from HPLC-resolved metabolites, the low-abundance metabolic intermediates, such as benzyl alcohol, benzaldehyde, benzoic acid, catechol, *cis*,*cis*-muconic acid, butanol, butanal, and butanoic acid, were detected by DI-ESI-HRMS analysis from the BBP-transforming resting-cell culture (1 h) using BBP-grown cells of strain PAE-6 (see Fig. S1 in the supplemental material).

### Respirometric assay.

The metabolic competence of various possible intermediates of BBP that stimulated oxygen uptake by washed cells of strain PAE-6 grown on BBP and its possible metabolic intermediates was appraised polarographically (Table 2). The molecular-oxygen-dependent metabolism of BBP-grown cells was reflected in the oxygen uptake profile in the presence of BBP, MBP, butanol, MBzP, benzyl alcohol, benzaldehyde, benzoic acid, and catechol with respect to that of cellular respiration. Regarding PA metabolic status as stated above, the cells displayed a very small level of oxygen uptake in the presence of PA compared to that of cells alone. Further, the oxygen uptake data, as revealed for benzyl alcohol, benzaldehyde, benzoic acid (BA), and catechol with benzyl alcohol-grown cells, support the metabolism of benzyl alcohol to tricarboxylic acid (TCA) cycle intermediates via BA and catechol, where the last two intermediates were supposed to be metabolized by oxygen-dependent ring-hydroxylating dioxygenase and ring cleavage dioxygenase, respectively. Again, the butanol-grown cells of strain PAE-6 showed oxygen uptake in the presence of butanol but not in the presence of butanal or butanoic acid, indicating the possible involvement of an oxygen-dependent alcohol oxidase in the metabolism of butanol.

**TABLE 2:** Oxygen uptake rates with various compounds by resting-cell transformation of *Rhodococcus* sp. strain PAE-6 grown on different substrates^*a*^

Assay substrate	Oxygen uptake rate (nmol O_2_ consumed min^−1^ mg^−1^) by cells grown on:			
	BBP	Benzyl alcohol	Butanol	Succinate
BBP	22.46	ND	ND	ND
MBP	21.78	ND	1.34	ND
MBzP	23.12	1.79	ND	ND
PA	3.61	ND	ND	ND
PCA	36.72	ND	ND	ND
Benzyl alcohol	37.83	37.45	ND	ND
BA	38.01	37.92	ND	ND
Catechol	38.82	38.96	ND	ND
Butanol	34.56	ND	35.36	ND

a All values are corrected for endogenous O_2_ uptake. ND, not detected.

On the other hand, the benzyl alcohol or butanol-grown cells did not show oxygen uptake in the presence of BBP, MBP, or MBzP. Oxygen uptake was also not observed in the presence of butanol with benzyl alcohol-grown cells or in the presence of benzyl alcohol, benzaldehyde, BA, or catechol with butanol-grown cells of strain PAE-6. Moreover, none of the molecular oxygen-consuming metabolic activities mentioned above was observed with succinate-grown cells, indicating the inducible nature of the BBP catabolic pathway and possible involvement of multiple inducible catabolic operons. It is important to mention that some of the compounds, such as BBP, MBP, MBzP, benzyl alcohol, and benzaldehyde, are not directly metabolized by an oxygen-dependent enzyme; however, the observed *in vivo* oxygen uptake profiles in the presence of these compounds are due to the oxygen-dependent metabolism of their metabolic intermediates, namely, benzoic acid, catechol, and butanol.

### Enzyme assays.

**FIG 4:**
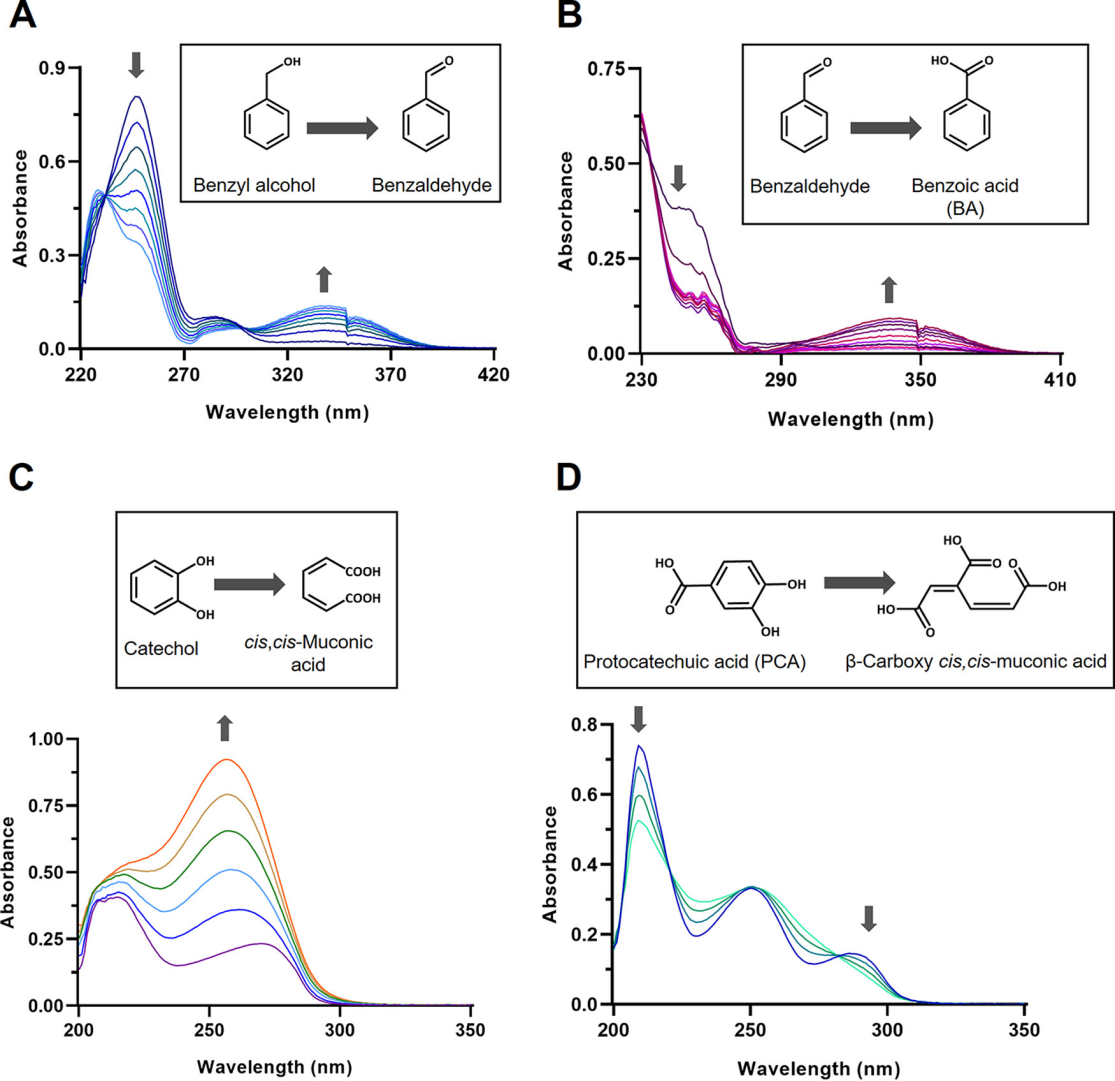
Evaluation of enzyme activity using spectral changes of substrate. Transformation of benzyl alcohol (A), benzaldehyde (B), catechol (C), and PCA (D) by the cell extract of BBP-grown cells of strain PAE-6. The sample and reference cuvettes contained 50 mM potassium phosphate buffer (pH 7.0) and 150 nmol of NAD^+^ in a 1-mL volume (A and B). The sample cuvette also contained 270 nmol of benzyl alcohol (A) and 60 nmol of benzaldehyde (B), while spectra were recorded at 2-min intervals up to 16 min (A) and 20 min (B) after the addition of 50 μg of protein to both cuvettes. For the transformation of catechol and PCA, the sample and reference cuvettes contained 50 mM potassium phosphate buffer (pH 7.0) in a 1-mL final volume. The sample cuvette contained 180 nmol of catechol (C) and 100 nmol of PCA (D), and spectra were recorded at 2-min intervals up to 12 min (C) and at 0, 3, 6 and 9 min (D) after the addition of 50 μg crude protein to both cuvettes. Up and down arrows indicate increasing and decreasing absorbance, respectively.

**(i) Metabolism of benzyl alcohol.** Transformation of benzyl alcohol by cell extracts of strain PAE-6 grown on BBP, MBzP, or benzyl alcohol in the presence of NAD^+^ showed a time-dependent gradual decrease in absorbance of the spectrum around 260 nm with a concomitant increase in absorbance around 340 nm, indicating the transformation of benzyl alcohol to benzaldehyde by a NAD^+^-dependent dehydrogenase with the formation of NADH (λ_max_ 340 nm) (Fig. 4A) (14). At the same time, Fig. 4B illustrates the NAD^+^-dependent enzymatic transformation of benzaldehyde to BA by the same cell extracts, depicting a characteristic decrease in *A*_248_ and an increase in *A*_340_ (formation of NADH) (14). Such spectral changes were not observed in the extracts of the cells of strain PAE-6 grown on MBP, butanol, or succinate. These results suggest the involvement of an inducible NAD^+^-dependent dehydrogenase(s) in the transformation of benzyl alcohol to BA via benzaldehyde in strain PAE-6.

**(ii) Metabolism of catechol.** A shift in the absorption maximum of 275 nm of catechol to 260 nm was noticed during the transformation of catechol by the extracts of cells grown on BBP, MBzP, benzyl alcohol, or BA, indicating the *ortho*-cleavage of catechol by catechol 1,2-dioxygenase with the formation of *cis*,*cis*-muconic acid (Fig. 4C). On prolonged incubation of the reaction mixture, a decrease in *A*_260_ was observed, indicating the possible metabolism of *cis*,*cis*-muconic acid to muconolactone, which has a lower extinction coefficient at 260 nm than *cis*,*cis*-muconic acid (15). Thus, it may be concluded that benzyl alcohol is likely to be assimilated via benzaldehyde, BA, catechol, *cis*,*cis*-muconic acid, and muconolactone, leading to the TCA cycle intermediates. No such ring cleavage activity was observed in cell extracts obtained from MBP-, butanol-, or succinate-grown cells.

**(iii) Metabolism of PCA.** Transformation of protocatechuic acid (PCA) by the cell extract of strain PAE-6 grown on BBP revealed a characteristic decrease in the absorption at 290 nm in the optical spectra, furnishing evidence of *ortho*-cleavage of PCA by PCA-3,4-dioxygenase with the formation of β-carboxy-*cis*,*cis*-muconate (Fig. 4D) (16). Similar changes were also observed with the extracts of the cells of strain PAE-6 grown on MBP, MBzP, and PA supplemented with succinate or PCA, but no such conversion of PCA was observed in the extracts of benzyl alcohol-, butanol-, or succinate-grown cells. To validate the enzymatic transformation, β-carboxy-*cis*,*cis*-muconate was detected in the DI-ESI-HRMS analysis of the organic extract of the cell extract-mediated PCA-transforming reaction mixture (Fig. S2).

**(iv) Metabolism of butanol and butanal.** NAD^+^-dependent alcohol and aldehyde dehydrogenases are the common enzymes reported for the metabolism of butanol to butanoic acid via butanal (17). In the present study, no NAD^+^-dependent butanol dehydrogenase activity was detected. Instead, consumption of molecular oxygen was noticed polarographically in the presence of butanol as the substrate with BBP-, MBP- or butanol-grown cells of strain PAE-6, indicating a possible role of a NAD^+^-independent alcohol dehydrogenase or an alcohol oxidase (18). Involvement of an alcohol oxidase or a NAD(P)^+^-independent alcohol dehydrogenase in the transformation of butanol by extracts of BBP-, MBP- or butanol-grown cell was validated colorimetrically by measuring the formation of hydrogen peroxide (H_2_O_2_) based on *A*_555_ evaluation from the developed red color of the assay mixture (19). Further, alcohol oxidase activity was reinforced by dichlorophenolindophenol (DCPIP) assay using the same set of cell extracts and by measuring the change in color (optical density at 600 nm [OD_600_] of the reaction mixtures with respect to controls [20]), as depicted in Fig. S3. Thus, the above findings support the fact that butanol is metabolized by an inducible NAD(P)^+^-independent alcohol dehydrogenase (alcohol oxidase).

On the other hand, the transformation of butanal using the same set of cell extracts in the presence of NAD^+^ showed a gradual decrease in absorbance around 252 nm and a concomitant increase in absorbance around 340 nm with time (data not shown). This result indicated the transformation of butanal by a NAD^+^-dependent dehydrogenase to butanoic acid with the formation of NADH (λ_max_ = 340 nm) (14). Thus, butanol, one of the hydrolyzed products of BBP (Fig. S1), is transformed to butanoic acid via butanal by distinct catabolic enzymes involving an oxygen-dependent, NAD(P)^+^-independent alcohol dehydrogenase/alcohol oxidase followed by a NAD^+^-dependent dehydrogenase.

### Overview of the *Rhodococcus* sp. strain PAE-6 genome.

The draft genome sequence of strain PAE-6 resulted in a single chromosome of 5,636,515 bp with a GC content of 67.6%. Circular visualization of the genome is depicted in Fig. S4A. Analysis of the genome revealed the presence of 5,743 predicted protein-coding genes (coding sequences [CDS]), of which 3,582 proteins could be functionally assigned, apart from the occurrence of 168 tRNA and 12 rRNA operons. The identified CDS could be assigned to 27 categories of clusters of orthologous groups (COGs), suggesting that the organism is efficient in the metabolism of lipids, carbohydrates, amino acids, and various aromatic compounds (Fig. S4B). The whole-genome sequence data and NCBI-BLAST search results revealed the putative functions of most individual genes that are possibly responsible for the metabolism of BBP and its metabolic intermediates. In hydrolytic metabolism of PAEs, 16, 5, and 13 genes were identified from the genome data of strain PAE-6. They were annotated as putative esterase, carboxylesterase, and hydrolase (alpha/beta hydrolase), respectively. Their identities and the most similar gene products are depicted in Table S1.

### Transcriptional and proteomic analysis of genes/enzymes of BBP-degradative pathways.

**FIG 5:**
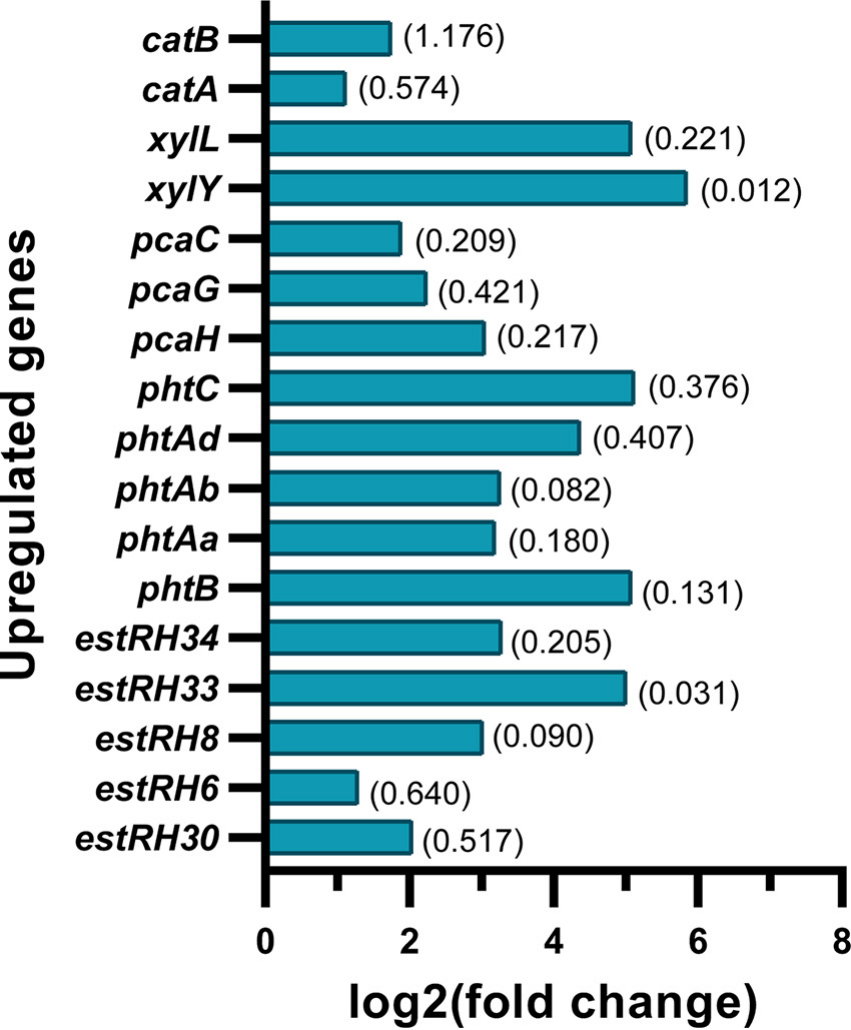
Transcriptome analysis representing differentially expressed genes in BBP-grown PAE-6 cells in comparison to the succinate-grown culture as control. The fold changes of upregulated *pht*, *pca*, *cat*, and *est* genes are depicted, with the corresponding *P* values shown in parentheses.

Transcriptome analysis revealed upregulation of specific relevant catabolic genes, as depicted in Fig. 5, along with their log fold change (FC) values compared to those obtained from succinate-grown culture. In elucidating genes involved in the inducible pathways of BBP degradation, among the esterase genes, it appeared that *estRH33* and *estRH34* showed maximum log FC values of 5.00 (*P* = 0.031) and 3.270 (*P* = 0.205), respectively. Figure S5 depicts the phylogenetic affiliation of the esterases EstRH33 and EstRH34 in the superfamily of esterase. Phthalate esterase EstRH34, affiliated with the mono-2-ethylhexyl phthalate (MEHP) hydrolase family, showed 100% identity with MEHP hydrolase (accession no. AB214635) from *Gordonia* sp. strain P8219 (21), while EstRH33 was affiliated with an unknown family of esterase, showing 80.18 and 77% identity with phthalate ester hydrolases (accession no. AAK16532 and ABG99214) from Arthrobacter keyseri 12B and Rhodococcus jostii RHA1, respectively (11). Further, both the lone phthalic acid-degrading *pht* operon and the protocatechuic acid-degrading *pca* operon showed upregulation in transcriptome analysis. Moreover, the benzoate- and catechol-metabolizing *xyl* and *cat* operons were also differentially expressed with changes in the range of 1.114- to 5.847-fold in the BBP-grown culture. All these transcriptome data indicate the induction of specific genome-borne genes/operons by BBP or its metabolites in the degradation process.

**FIG 6:**
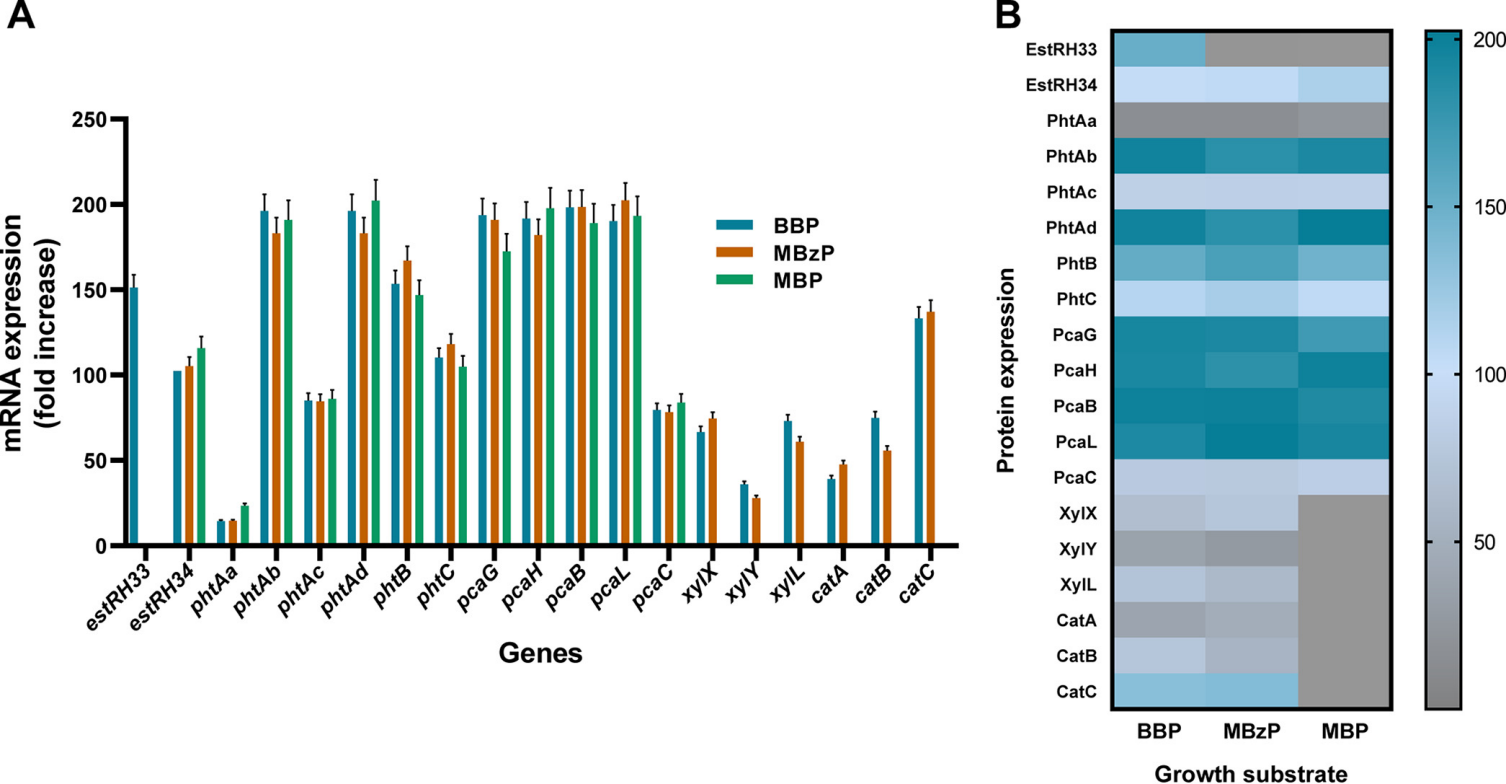
(A) RT-qPCR analysis of mRNA transcripts of BBP-degrading catabolic genes obtained from BBP-grown cells of strain PAE-6. Relative changes in gene expression were depicted with succinate-grown cells as controls, normalized to 16S rRNA as the endogenous control. Mean values were obtained from triplicate measurements. (B) Heat map profile of differentially expressed proteins analyzed by ESI-LC-MS in BBP-, MBzP-, and MBP-grown cultures of strain PAE-6 with respect to that of succinate-grown culture as control.

**FIG 7:**
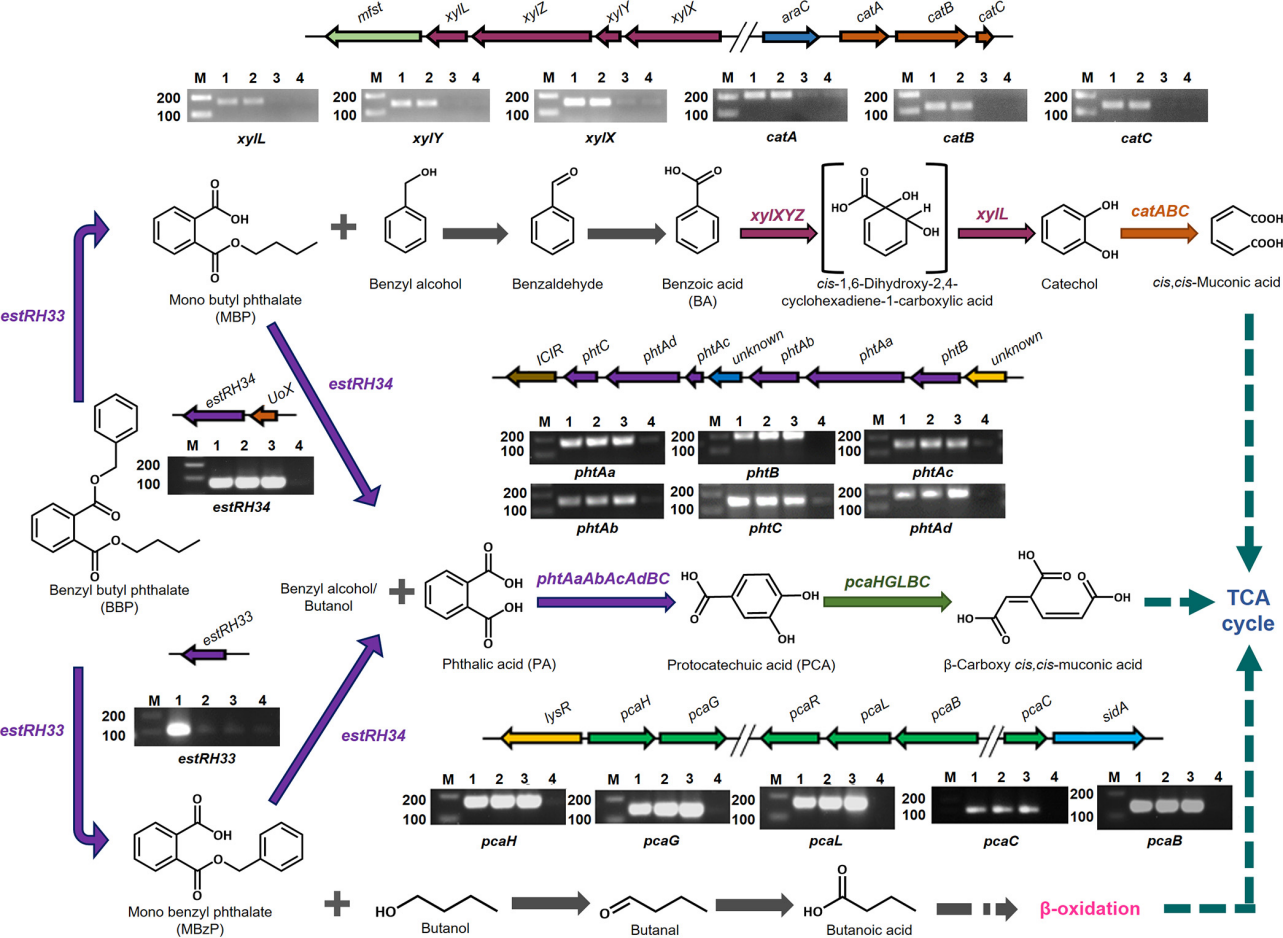
Metabolic pathway(s) of BBP in *Rhodococcus* sp. strain PAE-6, illustrating various inducible catabolic operons, and agarose gel electropherogram images of the overexpressed mRNA profiles, as determined by RT-qPCR analysis. Lanes 1 to 4 represent BBP-, MBzP-, MBP-, and succinate-grown cultures of strain PAE-6, respectively; lanes M contain molecular markers.

To confirm the transcriptome data above, reverse transcription-quantitative PCR (RT-qPCR) analysis was done independently from RNA isolated from the cultures of strain PAE-6 grown on BBP, MBzP, MBP, and PA supplemented with succinate or on succinate alone. The RT-qPCR study revealed that among the upregulated catabolic genes that were obtained from transcriptome analysis, six phthalic acid-metabolizing genes of the *pht* gene cluster (*phtBAaAbAcAdC*), five PCA-degrading genes of the *pca* gene cluster (*pcaHGBCL*), two esterase genes (*estRH33* and *estRH34*), and six benzoate- and catechol-degrading genes of the *xyl* and *cat* gene clusters (*xylLYX* and *catABC*) in BBP-grown cells were upregulated (Fig. 6A), reflecting the differential mRNA expression ratios. However, no such amplified gene products were observed when succinate was used as a growth substrate. The transcriptome and RT-qPCR results were further validated by proteomics data obtained using BBP-, MBzP-, MBP-, and succinate-grown cells (Fig. 6B). The metabolic pathway of the degradation of BBP, based on the biochemical and molecular analyses reported above, is illustrated in Fig. 7.

### Complete mineralization of BBP by coculture.

**FIG 8:**
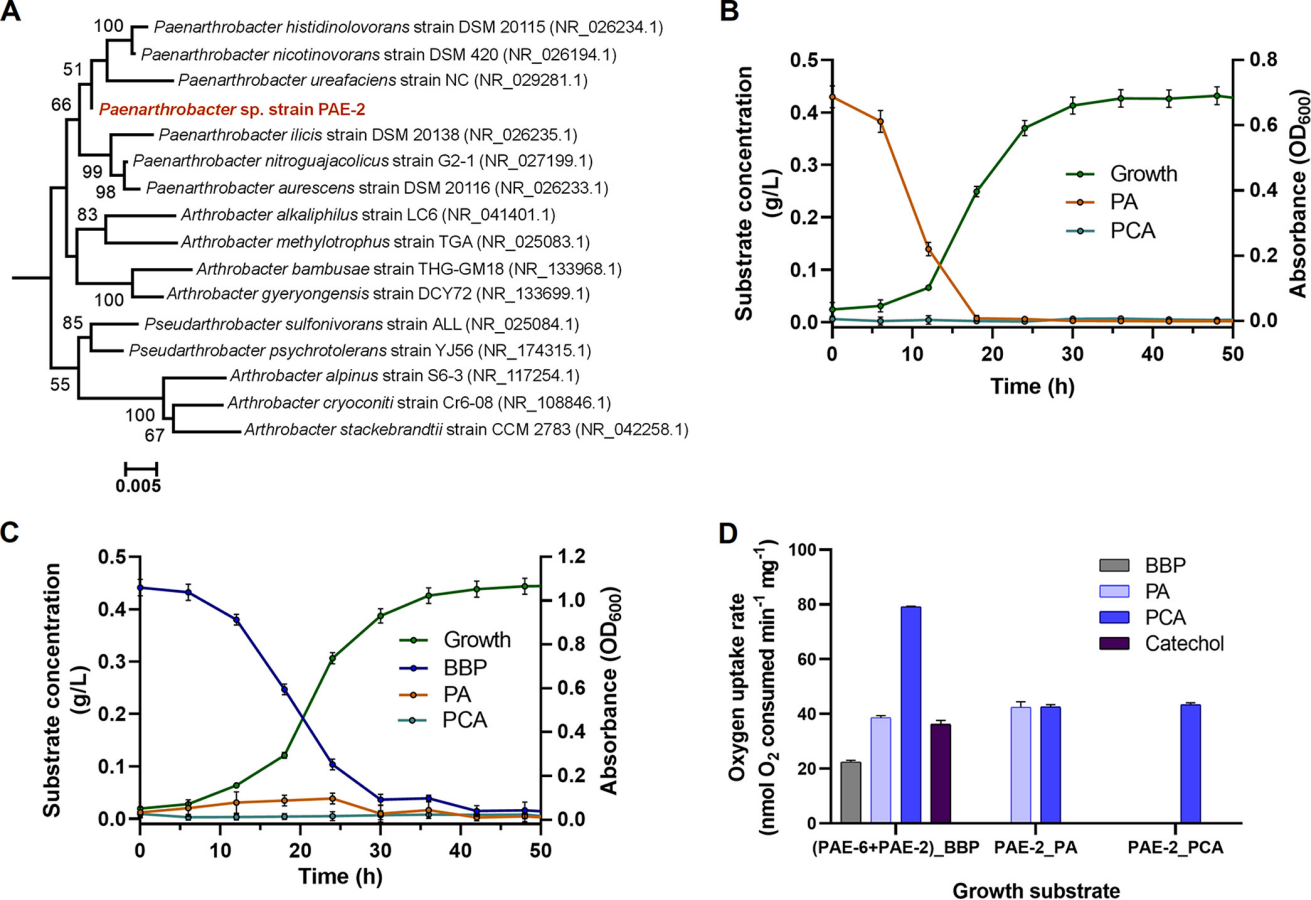
(A) Phylogenetic relationship based on 16S rRNA gene sequences of PAE-6 and representative strains of homologous genera. Numbers at the nodes indicate the levels of bootstrap support based on neighbor joining analysis of 100 resampled data sets. Bootstrap values below 50% are not shown. The scale bar represents 0.005 substitution per nucleotide position. GenBank accession numbers of the sequences are given in parentheses. The multiple sequence alignment was performed using ClustalX2 and the phylogenetic tree was constructed using neighbor joining algorithm as implemented in Tree Explorer 2.12. Strain PAE-2 is indicated in boldface type. (B and C) Growth of strain PAE-2 upon utilization of PA as the substrate (B) and growth of the coculture of PAE-6 and PAE-2 upon utilization of BBP (C); the status of the accumulation of metabolites is marked with different-colored lines. Vertical bars represent SD of mean values from triplicate measurements. (D) Oxygen uptake rates from resting-cell incubation of strain PAE-2 and coculture of PAE-6 and PAE-2 separately, grown on different substrates in the presence of various compounds.

In the present study, strain PAE-2, isolated from a BBP-enriched culture in association with strain PAE-6, efficiently utilized PA as the sole carbon source but not BBP. Analysis of the amplified 16S rRNA sequences (1,330 bp) and that obtained from whole-genome data (data not shown) revealed that strain PAE-2 belongs to the genus *Paenarthrobacter* and has a genome size of 4,512,836 bp and a GC content of 63.43%. In the NCBI database, Paenarthrobacter ureafaciens strain NC showed a maximum identity of 99.14% to PAE-2 at the 16S rRNA level. A phylogenetic tree of strain PAE-2 with the closest neighbors in terms of 16S rRNA gene sequence similarity is shown in Fig. 8A. Interestingly, strain PAE-2 could completely utilize PA (0.5 g L^−1^) within 30 h, and the growth rate was found to be 0.115 h^−1^ (Fig. 8B). Strain PAE-2 could also utilize PCA but not BBP, benzyl alcohol, benzoic acid, catechol, or butanol. Using an inoculum concentration of 4% (vol/vol) (OD_600_, 1.0) of both PAE-6 and PAE-2 in BBP-MSM (0.5 g L^−1^), the coculture could completely mineralize BBP in 38 h without any accumulation of PA (Fig. 8C). In addition, the PA-grown cells of strain PAE-2 showed oxygen uptake in the presence of PA and of PCA, but no oxygen uptake was observed in the presence of BBP, benzoate, or catechol (Fig. 8D). This supports the fact that strain PAE-2 cannot metabolize BBP and is also deficient in metabolizing benzoate and catechol. However, as expected, BBP-grown cells of the coculture of strains PAE-6 and PAE-2 showed positive oxygen consumption in the presence of BBP, PA, PCA, and catechol (Fig. 8D). Thus, in the degradation of BBP by the coculture, the inadequacy of PA metabolism in PAE-6 was alleviated, and complete assimilation of BBP was achieved.

### Possible molecular insight into inefficient metabolism of phthalic acid in strain PAE-6.

Regarding the metabolism of PA, phthalate 3,4-dioxygenases are predominant in actinobacteria, while phthalate 4,5-dioxygenases are predominant in proteobacteria. In actinobacteria, the *pht* gene cluster responsible for the metabolism of PA to PCA consisted of six functional genes (*phtAaAbAcAdBC*) and an additional open reading frame (ORF) (*phtU*) of unknown function. An analogous *pht* gene cluster was detected in the genome of strain PAE-6, but the strain could not efficiently utilize or transform PA. However, the gene cluster was found to be upregulated in BBP-, MBP-, MBzP-, and PA+succinate-grown cultures (Fig. 7 and Fig. S6), indicating that PA is the inducer of the operon. A very low level of utilization of PA by strain PAE-6 was reflected in the growth of the test organism individually in the presence of PA, PA+succinate, and succinate alone (Fig. S7). However, due to a very low level of transformation of PA to PCA and a possible rapid transformation of PCA to β-carboxy-*cis*,*cis*-muconate, PCA was supposed to be one of the transient metabolites. To reveal a relatively enhanced production of PCA, silver nitrate (AgNO_3_), an inhibitor of protocatechuate 3,4-dioxygenase (22), was used at a concentration of 1.0 μM in the resting-cell transformation of PA (0.5 g L^−1^) by strain PAE-6, grown in the presence of BBP or PA+succinate for the accumulation of PCA, which was detected by DI-ESI-MS (Fig. S8).

**FIG 9:**
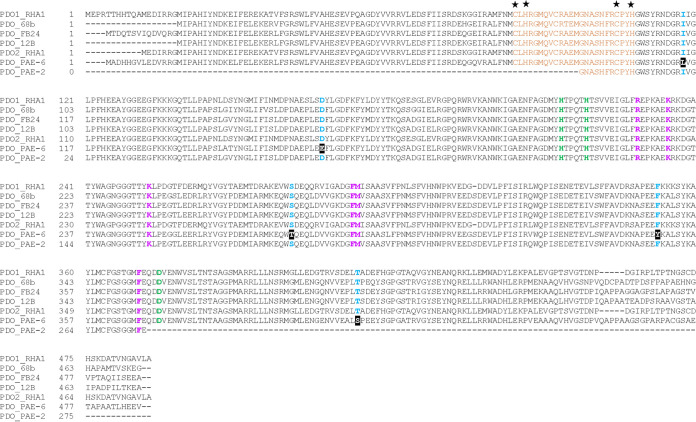
Multiple sequence alignment of α-subunits of PDO of strains PAE-6 and PAE-2 and other homologous PDO sequences. The consensus (CXHX_16-17_CXXH) residues for the binding of Rieske-type 2Fe-2S centers are in orange and marked with asterisks, while the conserved residues that coordinate the mononuclear iron are in green boldface type. The conserved amino acid residues Arg, Phe, Lys, and Met that are involved in substrate binding in 3,4-PDOs (23) are in purple boldface type. Other exclusively altered residues in PDO_PAE-6 among the conserved residues in blue are white on a black background. The accession numbers of the sequences used in the multiple sequence alignment are YP_707370.1 (PDO1_Rhodococcus jostii RHA1), AFM73571.1 (PDO_*Arthrobacter* sp. 68b), YP_829381.1 (PDO_*Arthrobacter* sp. FB24), AAK16534.1 (PDO_*Arthrobacter keyseri*), ABH00401.1 (PDO2_Rhodococcus jostii RHA1), MCT7294177.1 (PDO_*Rhodococcus* sp. PAE-6), and MCW3768948.1 (PDO_*Paenarthrobacter* sp. PAE-2).

Thus, it appeared that phthalate 3,4-dioxygenase is possibly the bottleneck in the poor metabolism of PA, since no accumulation of 3,4-dihydro-3,4-dihydroxyphthalate (or its abiotically dehydrated product, hydroxyphthalate) or its dehydrogenated product, 3,4-dihydroxyphthalate, could be detected upon the resting-cell transformation of PA by strain PAE-6, grown in the presence of BBP or PA+succinate. Figure 9 illustrates the multiple-sequence alignment of PhtAa of strain PAE-6, strain PAE-2, and other homologous phthalate 3,4-dioxygenases, displaying a 2Fe-2S binding domain, mononuclear iron-binding residues, reported substrate-interacting residues (23), and the presence of exclusively altered residues in the PDO obtained from strain PAE-6. Further, the possible impact of the altered residues was evaluated from the structural differences of PhtAa and its *in silico* mutant (restoring the modified residues) while comparing with the biochemically characterized α subunit of PDO-2 (PadAa) from Rhodococcus jostii RHA1 (24) (Fig. S9) using the modeling program AlphaFold2 (25). Below, we discuss the differences at the sequence level of the α subunit of phthalate dioxygenase and its possible role in catalysis.

## DISCUSSION

The genus *Rhodococcus*, a phylogenetically diverse group within nocardioform actinomycetes, can display diverse metabolic capabilities and degrade a wide variety of natural organic and xenobiotic compounds, including PAEs (26, 27). In the context of degradation of BBP, *Rhodococcus* sp. strain HS-D2, isolated from river sediment, was shown to utilize BBP, and the presence of MEHP hydrolase and PAE hydrolase, as revealed from genome data, was reported for the catalytic transformation of BBP to butanol, benzyl alcohol, and PA (28). A recent report illustrated microbial degradation pathways of DEHP in O_2_-limited estuarine sediments, suggesting side chain hydrolysis as the rate-limiting step of anaerobic DEHP degradation. An integrated *meta*-omics approach proposed that DEHP biodegradation in estuarine sediments is mainly achieved through synergistic metabolism by denitrifying proteobacteria using rate-limiting extracellular hydrolase to metabolize DEHP to PA, which is further metabolized by the UbiD-dependent benzoyl-coenzyme A (benzoyl-CoA) pathway (29). However, most actinobacterial species showed efficient aerobic hydrolytic metabolism of PAEs to PA and side chain alcohols; subsequently, PA is metabolized to PCA (30), and PCA is metabolized by either *ortho*-cleavage dioxygenase (present both in actinobacteria and proteobacteria) or *meta*-cleavage dioxygenase (exclusively in proteobacteria) pathway (31). On the other hand, in the anaerobic degradation pathway, in some denitrifying and sulfate-reducing bacteria, PA is activated by either type III CoA transferase or ATP-dependent CoA transferase to form highly unstable phthaloyl-CoA. This is further transformed to form benzoyl-CoA by nonoxidative decarboxylation using prenylated flavin mononucleotide (FMN)-dependent phthaloyl-CoA decarboxylase of the UbiD family and subsequently cleaved through the benzoyl-CoA degradation pathway (32, 33).

It should be mentioned here that apart from esterase/hydrolase, assimilation of BBP necessitates multiple catabolic genes and operons for the metabolism of PA-PCA, benzyl alcohol-benzaldehyde-BA-catechol, butanol-butanal-butyric acid and various central metabolites-TCA cycle intermediates. According to a recent report (34), most phthalate hydrolases belong to families IV, VI, VII, VIII, XVII, and MEHP hydrolase, except for a few that belong to other families or unknown categories of hydrolases (35–37). In the present study, BBP was hydrolyzed by EstRH33 to MBP and MBzP, which were further hydrolyzed by EstRH34. Moreover, *estRH33* was found to be upregulated only in the case of BBP-grown cells, signifying that BBP is the inducer of *estRH33* (diesterase). In contrast, *estRH34* was upregulated in the case of BBP-, MBP-, and MBzP-grown cells, indicating both MBP and MBzP are the inducers of *estRH34* (monoesterase). However, none of the hydrolases were upregulated in PA+succinate- or PCA-grown cells. On the other hand, the hydrolyzed benzyl alcohol was metabolized by dehydrogenase(s) to benzoic acid, which was further degraded by *xyl* and *cat* gene clusters. The RT-qPCR data also revealed that benzoate- and catechol-degrading gene clusters (*xyl* and *cat*) were upregulated in BBP-, MBzP-, benzyl alcohol-, and benzoate-grown cells but not in MBP-, PA+succinate-, PCA-, and butanol-grown cells, indicating that benzoic acid and catechol (or their metabolites) are the inducers of *xyl* and *cat* operons, respectively.

Additionally, RT-qPCR data also confirm the upregulation of *pht* and *pca* gene clusters for the degradation of BBP-hydrolyzed phthalic acid. However, the latter was found to be poorly utilized in strain PAE-6. Despite its being a poor degrader of PA, all the genes of the *pht* gene cluster responsible for the metabolism of PA were upregulated in BBP- and PA+succinate-grown cultures of strain PAE-6. Again, upregulation of the *pca* gene cluster was observed in BBP-, PA+succinate-, and PCA-grown cultures, indicating that PA and PCA (or their metabolites) are the inducers of the *pht* and *pca* gene clusters, respectively. This was supported by the enzymatic transformation of PCA to β-carboxy-*cis*,*cis*-muconate (similar to that shown in Fig. 4D) by the cell extracts of PA+succinate- and PCA-grown cultures.

Among the six functional genes (*phtAaAbAcAdBC*) of the *pht* operon, *phtAaAbAcAd* encode a multicomponent dioxygenase which can transform PA to 3,4-dihydro-3,4-dihydroxyphthalate. The dihydrodiol is further metabolized by a dehydrogenase (PhtB) to 3,4-dihydroxyphthalate and finally transformed to PCA by a decarboxylase (PhtC) (38). The multicomponent dioxygenase (Rieske oxygenase) encoded by *phtAaAbAcAd* of strain PAE-6 consists of an oxygenase α-subunit (*phtAa*), a β-subunit (*phtAb*), a ferredoxin (*phtAc*), and a reductase (*phtAd*). The reductase component of a functional ring-hydroxylating dioxygenase transfers electrons from NADP(H) to the ferredoxin component, which then transfers electrons to the mononuclear iron of the oxygenase α-subunit via its Rieske-type iron-sulfur cluster (2Fe-2S) (39). Based on sequence similarity studies with analogous components of PDOs, functionally related signature residues for both ferredoxin and reductase components of *phtAaABAcAd*, present in strain PAE-6, were found to remain conserved (data not shown). Again, in the case of the catalytic α-subunit (PhtAa) of strain PAE-6, a basic conservation of residues was observed in multiple-sequence alignment of the α-subunits of various reported phthalate 3,4- and 4,5-dioxygenases (data not shown), exhibiting the presence of a consensus sequence (CXHX_16-17_CXXH) for the binding of Rieske-type 2Fe-2S centers (40), besides two histidines (His) and one aspartate (Asp) residues for the binding of mononuclear iron (23). In the α-subunit of phthalate 3,4-dioxygenase, among the substrate interacting residues, Arg218 interacts with the C2 carboxylate group of PA, Met283 confers steric hindrance to prevent 4,5-dihydroxylation, Lys224 and Lys242 interact with the C1 carboxylate group of PA, and Phe282 and Phe359 impart π–π stacking interaction with the aromatic ring of the substrate for its regiospecific 3,4-dihydroxylation as revealed in the previous study with PDO_RHA1_ (23). Nevertheless, in the multiple-sequence alignments of α-subunits of phthalate 3,4-dioxygenase (Fig. 9), a few exclusive mismatches were found in the corresponding subunit of PDO from strain PAE-6. However, the aforesaid substrate-interacting catalytic pocket residues remain conserved. No such changes were observed in the α-subunit of PDO from PA-degrading strain PAE-2, as in that of other functional phthalate 3,4-dioxygenases (Fig. 9). Nonetheless, for optimum enzyme-ligand interaction, often, relatively distant residues about the catalytic pocket region as well as residues of tunnels and channels connecting to active site play an important role in efficient catalysis (41, 42). In the structural analysis using the modeling program AlphaFold2, no drastic structural changes were noticed, as the altered amino acids are similar to that of conserved amino acids (Fig. 9; Fig. S9). However, a comparison of PhtAa of strain PAE-6 and PadAa (PDO-2) of strain Rhodococcus jostii RHA1 revealed a change in the position of conserved Lys224 (substrate-interacting residue) and a structural difference due to the change of conserved Phe341 to Tyr349 (in PhtAa of strain PAE-6), apart from changes in the structure of various loops. These changes may have a possible impact on the poor functional activity of PDO_PAE-6_. On the other hand, a comparison of an *in silico* mutant of PhtAa with PadAa showed a better-aligned structure, restoring the majority of the changes (Fig. S9), which was supported by a decrease in root mean square deviation (RMSD) value from 0.456 Å (PadAa and PhtAa) to 0.395 Å (PadAa and PhtAa_mutant_).

Thus, the altered amino acid residue pattern may play a role(s) in inadequate binding or channeling of PA in the phthalate 3,4-dioxygenase of strain PAE-6, resulting in its poor *in vivo* metabolism. Nevertheless, this is the first encountered situation of poor metabolism of PA in a bacterial strain where the associated *pht* operon is upregulated in the presence of PA as an inducer molecule. In a future endeavor, the sequence information will be used in genome editing of *phtAa* to evaluate the functional potential of phthalate 3,4-dioxygenase in strain PAE-6 for efficient PA metabolism.

## MATERIALS AND METHODS

### Chemicals.

Benzyl butyl phthalate (BBP), monobenzyl phthalate (MBzP), mono-*n*-butyl phthalate (MBP), benzyl alcohol, 1-butanol, benzaldehyde, butanal, benzoic acid (BA), butyric acid, phthalic acid (PA), protocatechuic acid (PCA), and other phthalate diesters were purchased from Sigma-Aldrich GmbH (Germany). Catechol was purchased from Merck (Germany). Methanol, chloroform, and ethyl acetate, both analytical and HPLC grade, were purchased from Merck (India). All other chemicals and reagents used in this study were of analytical grade and used without further purification.

### Isolation and characterization of bacteria.

The strains used in this study were isolated from municipal-waste-contaminated soil at Dhapa, Kolkata (22.5373° N, 88.4334° E). The enrichment of cultures was started by inoculating a 1-g soil sample in liquid MSM (43) supplemented with BBP (0.5 g L^−1^) as the sole carbon and energy sources and incubated at 28°C on a rotary shaker (180 rpm). When growth was observed, the enrichment process was repeated with several transfers under the same conditions, and the enriched cultures were subsequently purified by plating on nutrient agar (2% [wt/vol]). For the identification of strains, the 16S rRNA gene was amplified by using f27 and r1492 as forward and reverse primers (44). The amplified product was sequenced according to the manufacturer’s specifications for *Taq* DNA polymerase-initiated cycle sequencing reactions using fluorescence-labeled dideoxynucleotide terminators with an ABI Prism 377 automated sequencer (Perkin-Elmer Applied Biosystems, Inc.). BLASTn of the National Center for Biotechnology Information was used to analyze the sequence homologies (45). The phylogenetic affiliations of the isolated strains were determined by comparing closely related 16S rRNA sequences using the neighbor-joining method (46) and ClustalX2 for sequence alignments. The tree was visualized and labeled using Tree Explorer 2.12. To study the cell morphology, cells of the log-phase culture of the test organism were grown individually in the presence of BBP and succinate and visualized under a scanning electron microscope (Quanta 200; FEI, USA). Before photographs were taken, cells were mounted on slides, fixed using 2.5% glutaraldehyde at 4°C for 1 h, and then dehydrated with a series of ethanol solutions (30, 50, 70, 80, 90, and 100% [vol/vol]), followed by gold coating of the dehydrated cells.

### Genome sequencing, assembly, and annotation.

The genomic DNA of test organisms was extracted using a genomic DNA extraction kit (Thermo Fisher Scientific, USA) following the manufacturer’s protocol. The extracted DNA was used for *de novo* whole-genome sequencing on an Illumina NovaSeq 6000 platform using the paired-end strategy. The *de novo* assembly was done with the SPADES_v3.15.0 assembler using high-quality processed reads (47). A final draft genome was used for genome annotation by employing the RAST server (48) and the RNAmmer 1.2 server (49). COG analysis was performed using the reversed position-specific BLAST analysis using NCBI COG (version 2/2/2011) on the prokaryotic protein database with an E value of 0.001. MS Excel was used to compare the output COG IDs (50). The circular genome of PAE-6 was visualized by using the web server Proksee (https://proksee.ca).

### Culture conditions and isolation of metabolites.

Isolated strains were grown in a rotary shaker at 28°C in a carbon-free MSM supplemented with BBP, MBP, MBzP, benzyl alcohol, benzaldehyde, BA, 1-butanol, butanal, butyric acid, PA, PCA, or succinate (0.5 g L^−1^) as the sole carbon source. For streaking, 1.8% agar-agar (Himedia) was used in MSM or Luria broth (LB), as needed. Individual pure cultures were used as inoculum (5% [vol/vol]; OD_600_ of 1.0) for the growth experiments. The growth of bacteria was monitored by recording the absorbance at 600 nm, and the growth rate was calculated from the log-phase cultures as reported earlier (51). In order to identify metabolic intermediates in the degradation process, resting-cell transformation was performed using the cells of late-log-phase culture, harvested by centrifugation (8,000 × *g*, 10 min), and washed twice with the same volume of potassium phosphate buffer (PPB; 50 mM, pH 7.0). The cells were then resuspended in PPB to give a final optical density of 1.0 at 600 nm and incubated in the presence of BBP or its possible intermediates (0.5 g L^−1^) for a period of up to 24 h at 28°C. After the desired incubation period, the spent cultures and the resting-cell cultures were extracted three times with an equal volume of ethyl acetate before and after acidification to pH 1.5 to 2.0 with 2 N hydrochloric acid. The combined extracts were then dried over anhydrous sodium sulfate and evaporated in a rotary evaporator (Rotavapor R-100; Buchi, Switzerland) under reduced pressure. The dried residue containing the unconverted substrate and metabolic intermediates was dissolved in a minimum volume of ethyl acetate for further analysis. Unless mentioned otherwise, all experiments were performed in triplicate.

### Preparation of cell extracts.

Cell extracts of isolated strains were prepared from the resuspended cells of late-log-phase cultures, as mentioned above, using a precooled French press (Constant cell disruption system, One Shot model; Constant Systems, Ltd., United Kingdom), following lysis of cells at 30,000 lb/in^2^ for two cycles. The extract was centrifuged at 30,000 × *g* for 20 min at 4°C to remove the cell debris. The supernatant obtained was used as the cell extract and subjected to protein estimation with bovine serum albumin as the standard (52).

### Analytical methods.

The organic extract of the spent culture or resting-cell culture containing the unconverted BBP and its metabolites was resolved by TLC on silica gel 60 GF_254_ plates (Merck, Germany) using the solvent system hexane-chloroform-glacial acetic acid (10:3:2) and was detected with a UV lamp at 254 nm. The identity of the resolved products was confirmed by comparing them with the reference compounds, which were developed identically. The organic extracts were also resolved by HPLC using a Shimadzu model LC20-AT pump system (Shimadzu Corp., Kyoto, Japan) equipped with a diode array model SIL-M20A detector and an analytical Agilent C_18_ reverse-phase column attached to a model SIL-20A autosampler. The biodegraded products were eluted with a programmed gradient of a solvent system at a flow rate of 1.0 mL min^−1^ and detected at 254 nm. The mobile phase consisted of methanol (solvent A) and water (solvent B; pH 3.0), and gradient elution was set up as follows: 50% A (0 to 5 min), 50 to 65% A (5 to 15 min), 65 to 50% A (15 to 25 min), 50 to 80% A (25 to 35 min), 80% A (35 to 45 min), 80 to 50% A (45 to 55 min), and 50% A (55 to 60 min). Metabolites were identified by comparing their retention times and UV-visible spectra with those of the authentic compounds analyzed under the same set of conditions. Again, the ethyl acetate-extracted samples were analyzed using DI-ESI-HRMS. Mass-spectral data were collected in a positive or negative ion mode on a Xevo-G2-Xs-QTof mass spectrometer (Waters, USA) operated in scan mode over a mass range of *m/z* 50 to 500. The capillary voltage was set at 3,000 V; the source and desolvation temperatures were 80°C and 250°C, respectively. The cone gas flow rate was 50 L h^−1^. The molecular weights (*m/z*) of the biodegraded metabolites were confirmed by comparing the *m/z* values of respective authentic standards analyzed under identical conditions.

### Respirometric analysis.

The uptake of dissolved molecular oxygen was studied in a reaction mixture (3.0 mL total volume) containing 200 μL cell suspension (25 mg [wet weight]), 500 μL substrate (0.5 g L^−1^ in water or saturated water solution), and 2.3 mL PPB (50 mM, pH 7.0) using a YSI model 5300A biological oxygen monitor (Yellow Springs Instrument Co., Yellow Springs, OH) fitted with a Clark-type polarographic oxygen electrode. The reaction was initiated at 25°C by adding assay substrate, and oxygen uptake was measured continuously for 10 min. The rate of oxygen uptake was expressed in nanomoles per minute per milligram of protein. The rates were corrected for cellular respiration or endogenous oxygen consumption.

### Enzyme assays.

A Cary 100 Bio UV–visible-spectrum spectrophotometer (Varian Australia Pvt., Ltd.) was employed to depict enzyme-catalyzed transformations of possible metabolic intermediates (PCA, catechol, benzyl alcohol, or benzaldehyde) of BBP using quartz cuvettes with a 1-cm path length. A reaction mixture containing substrate-induced cell extracts (as the source of enzyme) and the appropriate substrate or possible metabolite in the presence or absence of NAD^+^ as a cofactor was scanned at 1-min intervals in the wavelength range of 200 to 500 nm for 10 cycles. Varian Cary Win UV Scan application software was used to analyze the data.

For the oxidase assay, a 2,6-dichlorophenol indophenol (DCPIP) reduction assay was performed as described earlier (20). Briefly, in a final volume of 3 mL of reaction mixture containing 25 μL of 6.7 mM DCPIP, 50 μL of 20 mM phenazine methosulfate, and 30 μL of 1-butanol or 1-butanal in 80 mM Tris-HCl (pH 8.7) and cell extract (50 μg of protein) of strain PAE-6 grown in the presence of BBP, MBP, MBzP, 1-butanol, 1-butanol, or succinate were added to measure oxidase activity. The experiment was performed at 28°C, and the formation of color was estimated by recording the absorbance of the reaction mixture at 600 nm.

### Transcriptome, RT-qPCR, and proteome analyses.

The test organism was serially cultured in MSM supplemented with BBP several times to obtain a BBP-induced culture, and a parallel succinate-grown culture was used as a negative control. For each set, the harvested late-log-phase cells grown in MSM-BBP or MSM-succinate and washed three times with 50 mM PPB (pH 7.0) were used to isolate total RNA. Cell lysis was carried out using the Constant cell disruption system (One Shot model; Constant Systems, Ltd., United Kingdom) at 30,000 lb/in^2^ for one cycle, followed by a conventional TRIzol method for RNA isolation (53). Before and after DNase treatment (RNase-free DNase I; Thermo Scientific), RNA quality was checked by agarose gel electrophoresis, and quantitative estimation was done using a multimode reader (CLARIOstar Plus; BMG LABTECH, Germany). Samples with an *A*_260_/*A*_280_ ratio greater than 1.8 were used for downstream analysis. To avoid a possible false-positive result in RT-qPCR, removal of contaminated DNA from isolated RNA was confirmed from an agarose gel electrophoresis profile when no band of PCR-amplified products was visible, using RNA as a template. Further, to carry out an RNA sequencing experiment (RNA-seq), the above-mentioned RNA samples were analyzed using the Illumina platform. The adapter sequences and low-quality bases were trimmed in the preprocessing step using AdapterRemoval v2 (54). From the preprocessed reads, rRNA sequences were removed by aligning the reads with SILVA (55) and Rfam (56) database using GEM-Mapper v3 (57) and subsequent workflow using SAMtools-1.15.1 (58), BBMap version 38.57 (59), and in-house scripts followed by alignment to the corresponding whole-genome sequence of PAE-6 using STAR version 2.7.6a. Differential expression analysis was performed using the cuffdiff program of the (cufflinks package v2.2.1) (60). On the other side, to cross verify the transcriptome data, in-house RT-qPCR was performed. To accomplish this, single-stranded cDNA was synthesized from 1 μg of DNA-free RNA by using a RevertAid First Strand cDNA synthesis kit (Thermo Fisher) using the ProFlexPCR system (Applied Biosystems). RT-qPCR was carried out in QuantStudio 5 (Applied Biosystems) using 4 μL of 2× DyNAmoColorFlash SYBR green PCR master mix (Thermo Fisher), 2 μL (10 mM) of gene-specific RT primers (forward and reverse) as mentioned in Table S2, and 1 μL of cDNA template in a total volume of 10 μL made up with molecular biology-grade water (Hi-Media). The 16S rRNA gene was taken as an endogenous control; amplicon sizes were 100 to 300 bp for all the genes assessed. Relative gene expression was determined using the cycle threshold (ΔΔ*C_T_*) method and visualized by agarose gel electrophoresis.

For proteome analysis, cell extracts as mentioned above, were lyophilized using an Edwards lyophilizer and processed further for trypsin digestion, followed by ESI-LC-MS (61). A C_18_ BEH column (Agilent) was used for peptide separation, and a Waters Xero-G2-Xs-QTof mass spectrometer was used in positive ion mode for analyzing the peptides. Water (A) and acetonitrile (B) supplemented with 0.1% formic acid were used as mobile phase solvents in a gradient mode. The time programs used were 5% A for 0 to 30 min, 5 to 35% A for 30 to 45 min, 35 to 5% A for 45 to 60 min, and 0% A for 60 to 80 min. The flow rate was kept at 30 μL min^−1^. The output peptides with differential intensities were matched with the whole-genome sequence data of strain PAE-6 using the software Mass Lynx version 4.1 and Progenesis QIP for acquisition and processing, respectively.

### Data availability.

The whole-genome shotgun projects with respect to strain PAE-6 and PAE-2 were deposited in DDBJ/ENA/GenBank under the accession numbers JAOCLZ000000000 and JAPDNV010000000, respectively. The transcriptome data of PAE-6 were deposited in the NCBI database under the accession numbers SRX18014859 and SRX18014860 for BBP- and succinate-grown cells, respectively.
