# Supplementary material for: Biochemical and Multi-Omics Approaches To Obtain Molecular Insights into the Catabolism of the Plasticizer Benzyl Butyl Phthalate in Rhodococcus sp. Strain PAE-6
Source: Microbiol Spectr. 2023 Jun 15;11(4):e04801-22. doi: 10.1128/spectrum.04801-22 (PMC10434107; doi:10.1128/spectrum.04801-22)
Supplement: Supplemental file 1 — Supplemental material. Download spectrum.04801-22-s0001.pdf, PDF file, 2.0 MB [file spectrum.04801-22-s0001.pdf]

Supplementary Information

Biochemical and multi-omics approaches to unfold molecular insights of the catabolism of plasticizer benzyl butyl phthalate in *Rhodococcus* sp. strain PAE-6

Suman Basu, Rinita Dhar, Mousumi Bhattacharyya and Tapan K. Dutta

Department of Microbiology, Bose Institute, EN-80, Sector V, Salt Lake, Kolkata – 700091, West Bengal, India

Running title: Biodegradation of benzyl butyl phthalate

Address correspondence to Tapan K. Dutta, tapan@jcbose.ac.in.

TABLE S1 PAE degrading genes and ORFs identified from PAE-6 genome

Locus ID	Gene	Putative function	Protein length	Most similar gene products (organism, accession no.)	% Identity	Reference
MCT7289531	*estRH1*	Alpha/beta hydrolase	299	Alpha/beta hydrolase *(Microbispora triticiradicis*, RGA05404)	77.74	1
MCT7289890	*estRH2*	Alpha/beta hydrolase	345	Alpha/beta fold hydrolase (*Rhodococcus hoagie,* MBM4523444)	79.42	2
MCT7290255	*estRH3*	Alpha/beta hydrolase	347	Esterase *(Sulfobacillus acidophilus* DSM10332, AEW03609)	39	3
				DBP hydrolase (*Acinetobacter* sp. M673, JQ478494)	99	4
MCT7290277	*estRH4*	Alpha/beta hydrolase	352	Alpha/beta hydrolase (*Rhodococcus pyridinivorans*, QXF83741)	99.69	5
MCT7290680	*estRH5*	Carboxylesterase/lipase family protein	508	Carboxylesterase (*Rhodococcus opacus* M213, EKT80416)	66.05	6
MCT7290881	*estRH6*	Alpha/beta hydrolase	291	3-Oxoadipate enol-lactonase (*Rhodococcus opacus* PD630, EHI40149)	72.98	7
				3-Oxoadipate enol-lactonase (*Rhodococcusrhodochrous*, OOL31356)	79.72	8
MCT7290978	*estRH7*	Alpha/beta hydrolase	349	Alpha/beta hydrolase (*Nocardia* sp., MBC7301364)	81.38	9
MCT7291036	*estRH8*	Putative lipase	276	Esterase *(Sulfobacillus acidophilus* DSM10332, AEW03609)	24	3
				DBP hydrolase (*Acinetobacter* sp. M673, JQ478494)	94	4
MCT7291045	*estRH9*	Alpha/beta hydrolase	289	2-Hydroxy-6-ketonona-2,4-dienedoic acid hydrolase *(Rhodococcus* sp., AAF81825)	95.16	10
MCT7291103	*estRH10*	Alpha/beta hydrolase	279	Alpha/beta hydrolase (*Rhodococcus pyridinivorans*, WP_231414074)	98.92	11
MCT7291154	*estRH11*	Beta-lactamase family protein	419	Putative esterase (*Rhodococcus aetherivorans*, GES36575)	76.44	12
MCT7291184	*estRH12*	Beta-lactamase family protein	401	Class A beta-lactamase-related serine hydrolase (*Rhodococcus* sp., RZL82441)	65.03	13

MCT7291421	*estRH13*	Esterase family protein	332	Alpha/beta hydrolase-fold protein (*Rhodococcus pyridinivorans*, WP_254583585)	99.70	14
MCT7291532	*estRH14*	Alpha/beta hydrolase	269	Alpha/beta hydrolase-fold protein (*Rhodococcus rhodochrous,* WP_229579938)	95.56	11
MCT7291559	*estRH15*	Esterase family protein	439	Esterase (*Rhodococcus* sp. p52 AOD21404)	99.7	15
MCT7291730	*estRH16*	Alpha/beta hydrolase	283	Alpha/beta fold hydrolase (*Rhodococcus hoagie,* NKR27784)	97.17	16
MCT7291736	*estRH17*	Alpha/beta hydrolase	290	Alpha/beta fold hydrolase (*Rhodococcus hoagie,* NKS73933)	99.31	16
MCT7291872	*estRH18*	Alpha/beta hydrolase	303	Alpha/beta hydrolase fold domain-containing protein (*Rhodococcus pyridinivorans,* WP_248689755)	99.67	11
MCT7291950	*estRH19*	Hypothetical protein	115	Carboxylesterase (*Rhodococcus aetherivorans*, GES36833)	69	12
MCT7292253	*estRH20*	Esterase family protein	353	Esterase family protein (*Rhodococcus rhodochrous*, MCB8909510)	98.79	17
MCT7292295	*estRH21*	Esterase family protein	333	Alpha/beta hydrolase-fold protein (*Rhodococcus* sp. GA1 WP_255025845)	99.70	18
MCT7292317	*estRH22*	Alpha/beta hydrolase	352	Alpha/beta hydrolase (*Rhodococcus rhodochrous*, OOL29416)	78.90	8
MCT7292527	*estRH23*	Alpha/beta hydrolase	189	Esterase family protein (*Rhodococcus pyridinivorans* QXF82820)	96.03	5
MCT7292704	*estRH24*	Esterase family protein	339	Alpha/beta hydrolase-fold protein (*Rhodococcus pyridinivorans*, WP_252173058)	99.71	14
MCT7292708	*estRH25*	Alpha/beta hydrolase	285	Alpha/beta hydrolase (*Rhodococcus pyridinivorans*, WP_200358530)	96.84	11
MCT7293389	*estRH26*	Carboxylesterase/lipase family protein	512	Para-nitrobenzyl esterase (*Rhodococcus* sp. B50, MBS9373001)	92.58	19
MCT7293391	*estRH27*	Alpha/beta hydrolase	344	Carboxylesterase LipF (*Rhodococcus* sp. B50, MBS9373003)	87.61	19
MCT7293486	*estRH28*	Alpha/beta hydrolase	290	Carboxylesterase (*Rhodococcus* sp. WAY2, QHE72710)	33.82	20

MCT7293703	*estRH29*	Alpha/beta hydrolase	259	3-Oxoadipate enol-lactonase 2 (*Rhodococcus* sp. B50, MBS9373410)	97.68	19
MCT7293719	*estRH30*	Alpha/beta hydrolase	340	Esterase (*Rhodococcus erythropolis*, OXM23922)	58.5	21
MCT7293869	*estRH31*	Esterase family protein	362	Alpha/beta hydrolase-fold protein (*Rhodococcus rhodochrous,* WP_230792719)	98.25	18
MCT7294065	*estRH32*	Carboxylesterase/ lipase family protein	514	Para-Nitrobenzyl esterase (*Bacillus subtilis*, PDB: 1QE3_A)	39.16	22
MCT7294180	*estRH33*	N-carbamoylsarcosine amidohydrolase	218	Phthalate ester hydrolase (*Rhodococcus jostii* RHA1, ABG99214)	77	23
MCT7294181	*estRH34*	Phthalate monoesterase	304	MEHP hydrolase (*Gordonia* sp. P8219, AB214635)	100	24
MCT7294178	*phtB*	3,4-dihydroxy-3,4-dihydrophthalate dehydrogenase	288	Aldo/keto reductase (*Gordonia terrae* RL-JC02,CP049836.1)	100	25
MCT7294177	*phtAa*	phthalate 3,4-dioxygenase large subunit	487	Aromatic ring-hydroxylating dioxygenase subunit alpha (*Arthrobacter* sp. 68b, WP_173160822)	87.09	26
MCT7294176	*phtAb*	phthalate 3,4-dioxygenase small subunit	203	Phthalate 3,4-dioxygenase small subunit (*Gordonia* sp. HS-NH1, KM252678.1	76.08	27
MCT7294174	*phtAc*	Phthalate dioxygenase ferredoxin	65	Ferredoxin reductase (*Rhodococcus ruber* YC-YT1, CP023714.1)	100	28
MCT7294173	*phtAd*	Ferredoxin reductase	411	FAD-dependent oxidoreductase (*Arthrobacter* sp. FW306-05-C, WP_236800056)	75.91	29
MCT7294172	*phtC*	3,4-dihydroxyphthalate decarboxylase	249	3,4-Dihydroxyphthalate decarboxylase (*Terrabacter* sp. DBF63 ISTesp2, AB084235.1)	66	30
MCT7290033	*pcaH*	Protocatechuate 3,4-dioxygenase subunit beta	243	Protocatechuate 3,4-dioxygenase subunit beta (*Rhodococcus pyridinivorans* SB3094**,** CP006996.1)	100	31
MCT7290034	*pcaG*	Protocatechuate 3,4-	227	Protocatechuate 3,4-dioxygenase subunit alpha	100	31

		dioxygenase subunit alpha		(*Rhodococcus pyridinivorans* SB3094**,** CP006996.1)		
MCT7290950	*pcaB*	3-carboxy-cis, cis-muconatecycloisomerase	449	3-Carboxy-*cis, cis*-muconatecycloisomerase (*Rhodococcus pyridinivorans* SB3094**,** CP006996.1)	100	31
MCT7290949	*pcaL*	β Ketoadipate enol lactone hydrolase	390	3-Oxoadipate enol-lactone hydrolase (*Rhodococcus pyridinivorans* SB3094**,** CP006996.1)	100	31
MCT7290383	*pcaC*	4-carboxy muconolactone decarboxylase	266	4-Carboxymuconolactone decarboxylase (*Gordonia terrae* RL-JC02, QIK47848.1)	100	32
MCT7290914	*xylX*	Benzoate 1,2-dioxygenase subunit alpha	457	Benzoate dioxygenase large subunit (*Rhodococcus* sp. 19070,AAK58903.1)	90.97	33
MCT7290913	*xylY*	Benzoate 1,2-dioxygenase subunit beta	167	Benzoate 1,2-dioxygenase subunit beta (*Rhodococcus* sp. p52, CP016819.1)	100	15
MCT7290911	*xylL*	1,6-dihydroxycyclohexa-2,4-diene-1-carboxylate dehydrogenase	265	1,6-Dihydroxycyclohexa-2,4-diene-1-carboxylate dehydrogenase (*Rhodococcus rhodochrous strain* ATCC BAA870, CP032675.1)	100	34
MCT7290923	*catA*	Catechol 1,2-dioxygenase	283	Catechol 1,2-dioxygenase (*Rhodococcus pyridinivorans* SB3094**,** CP006996.1)	100	31
MCT7290924	*catB*	Muconate cycloisomerase	375	Muconate cycloisomerase *(Rhodococcus opacus*, X99622.2)	97	35
MCT7290925	*catC*	Muconolactone isomerase	93	Muconolactone delta-isomerase (*Rhodococcus pyridinivorans* SB3094**,** CP006996.1)	100	31

TABLE S2 RT-PCR primers

Genes	RT-PCR primer	Primer sequences (5' to 3')
estRH6	Forward	TCCTGTGTATCCACACTGCG
	Reverse	CGTAGGTGATCAGGCTCGTG
estRH8	Forward	GCTGTGGTAGGCGGTATGTT
	Reverse	CGGTCGAATTCCTCGTCCAT
estRH30	Forward	AGATGTCGGTATTGAACT
	Reverse	GATAGAACAACGCATCCA
estRH33	Forward	TGCCGTTGAAGAATACGAACG
	Reverse	GTACTTGTTGTCGATGTC
estRH34	Forward	CCCTGAGATGGTAAAGAA
	Reverse	GATAGTGACGGATCAACT
phtB	Forward	CATAGCATTCCGCATCTG
	Reverse	GTTGAACTTGCTGGAGAC
phtAa	Forward	GTGTCCCTCACCAATACT
	Reverse	TGGTTGTACTCGCTGTAG
phtAb	Forward	GAACAAGTACTCGCTCAG
	Reverse	AGTCCACGATGATCTCTT
phtAc	Forward	CGGAGCCTCTGACTACTT
	Reverse	TCATGCGTCCTCTACCAC
phtAd	Forward	CAACGAGTACTGTCAAGC
	Reverse	CGGATGGATGATGTTGTAG
phtC	Forward	ATGACACCCGGAAACTTG
	Reverse	GCAATGTGCTGTCGGTAA
pcaH	Forward	CCTGATCCTGAACTTCTC
	Reverse	GATAGGTGTCCTTCTTGT
pcaG	Forward	GACATCTTCGACAACAAC
	Reverse	GAAGTTCGGATCGAGATC
pcaB	Forward	CTGTTCGATCCCGTCTTC
	Reverse	GTGGGTGTTCTTGACACG
pcaC	Forward	ACTCCTACGAATTCGATCC
	Reverse	GTCGAAATGCTCGATGAC
XylX	Forward	TACATCATGGACCAGTTC
	Reverse	GTTGAAGAAGTCCTCGTA
XylY	Forward	CTGGTTCTCGTTGTACTT
	Reverse	GGATGTAGTCGTTCTTCA
XylL	Forward	AAGCCGTACGAGCACTAT
	Reverse	CGACGAGACGTTCACGAT
CatA	Forward	GACCGACAAGTTCAAGTC
	Reverse	GTGCTTCTGGATGATGTC
CatB	Forward	GCCTGAACTACTCCTTCAA
	Reverse	TTGACGTCGATGCTGAAT
CatC	Forward	GAAGGAGAAGGCCTACAG
	Reverse	CAGGATCTCGTGCAATTC
16S rRNA	Forward	CTAGTAATCGCAGATCAG
	Reverse	CTACCTTGTTACGACTTC

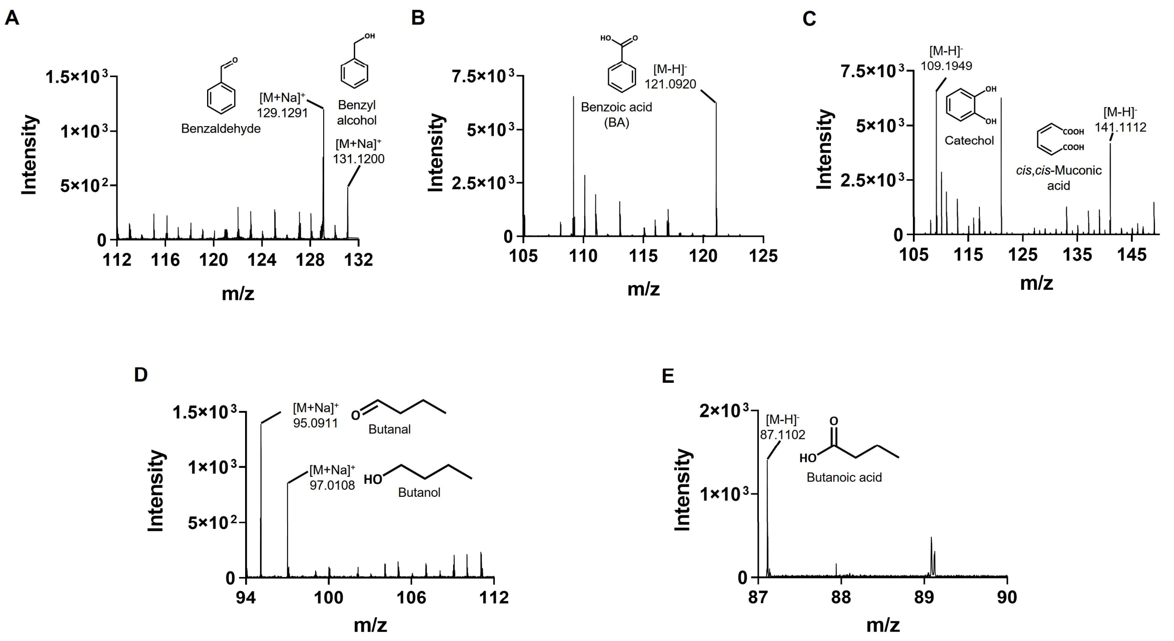

FIG S1 DI-ESI-HRMS analysis of organic extracts of resting cell culture of benzyl butyl phthalate (BBP)-grown cells of strain PAE-6 incubated in the presence of BBP for 1 h, showing the production of various metabolites, namely benzyl alcohol and benzaldehyde (A), benzoic acid (B), catechol and *cis*,*cis*-muconic acid (C), butanol and butanal (D) and butanoic acid (E).

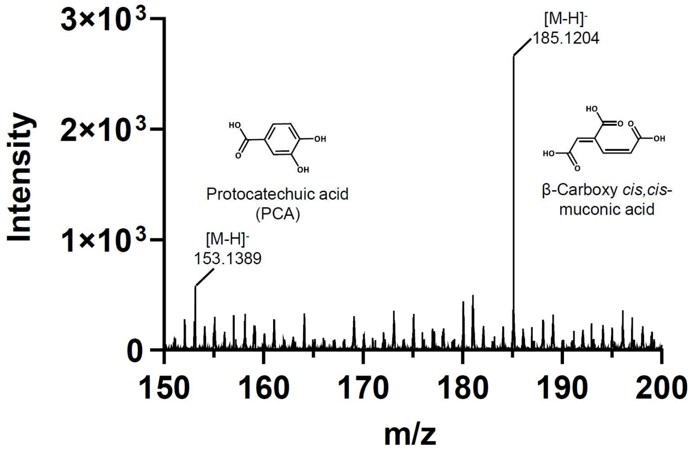

FIG S2 Identification of β-carboxy *cis,cis*-muconic acid by DI-ESI-HRMS analysis of the organic extract of the reaction mixture containing the cell-free extract (50 μg of protein) of BBP-grown cells of strain PAE-6 and protocatechuic acid (PCA), incubated for 10 min.

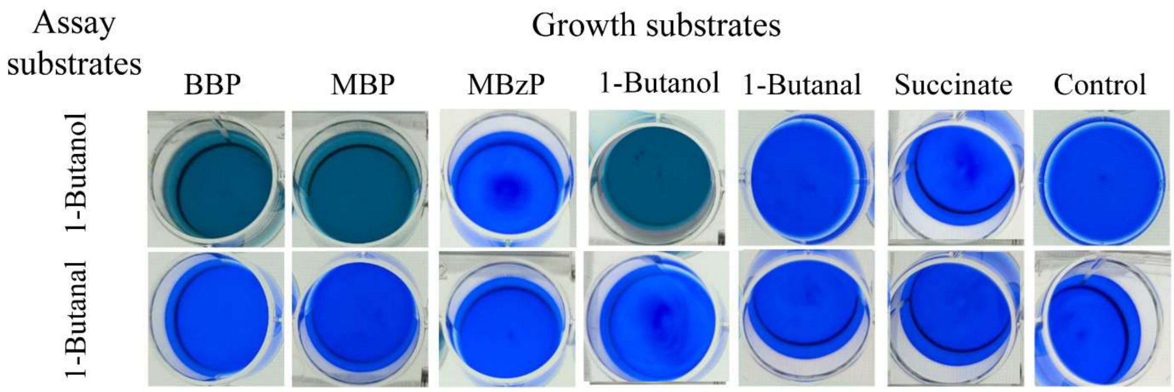

FIG S3 NAD(P)$^+$-independent dehydrogenases assay of 1-butanol (upper panel) and I-butanal (lower panel) with the cell-free extracts of strain PAE-6 grown individually in presence of various substrates using 2,6-dichlorophenol indophenols (DCPIP). Incubation of DCPIP alone was used as control.

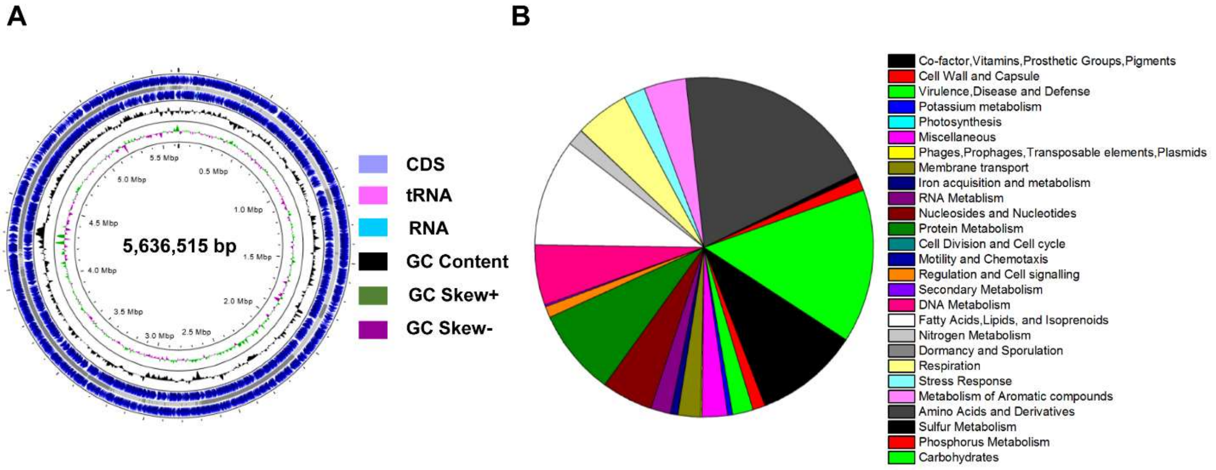

FIG S4 Circular visualization of draft genome of strain PAE-6 (left) and COG functional classification of encoded proteins in the genome (right).

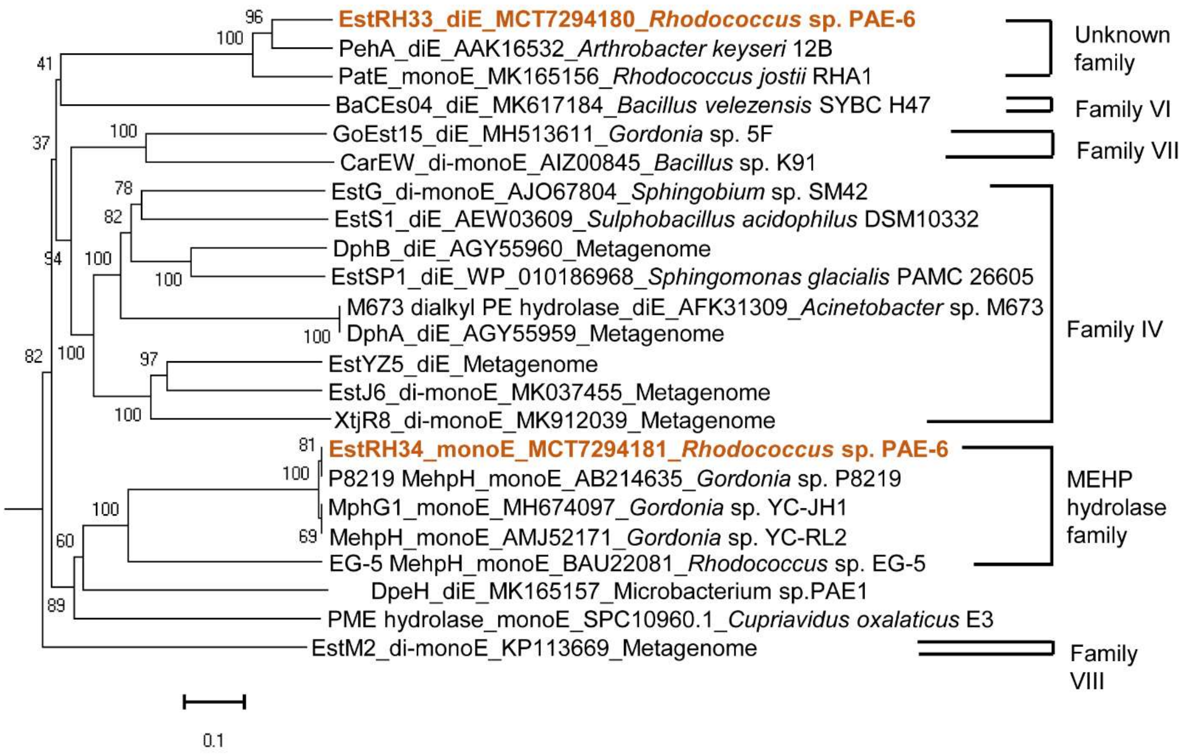

FIG S5 Phylogenetic relationship of phthalate hydrolases (*EstRH33* and *EstRH34*) of strain PAE-6 (shown in colored bold faces) with the reported phthalate hydrolases belonging to different esterase families. Numbers at the nodes indicate the levels of bootstrap support based on neighbour joining analysis of 100 resampled data sets. The scale bar represents 0.1 substitutions per amino acid position. GenBank accession numbers of the sequences are indicated within parentheses. The multiple sequence alignment was performed using ClustalX2 and the phylogenetic trees were constructed using neighbour joining algorithm as implemented in Tree Explorer 2.12.

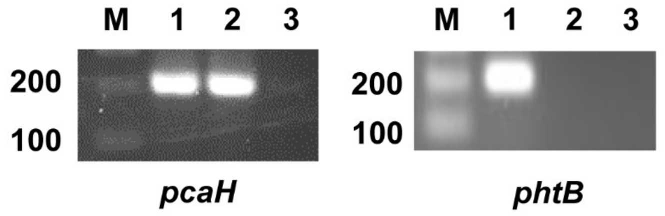

FIG S6 Agarose gel electropherogram images of the over-expressed mRNA profiles of *pcaH* and *phtB*, as determined by RT-qPCR analysis where 1, 2 and 3 represent phthalic acid (PA) + succinate, protocatechuic acid (PCA) and succinate-grown culture of strain PAE-6 while M stands for molecular marker.

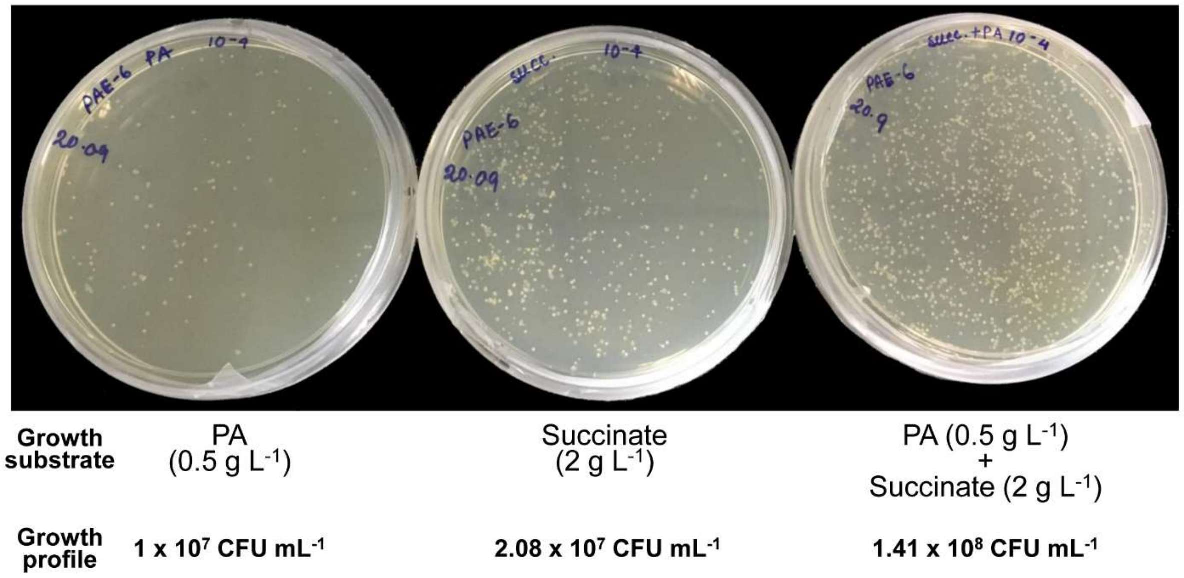

FIG S7 Growth profile of strain PAE-6 in presence of PA, succinate and PA+succinate as growth substrates.

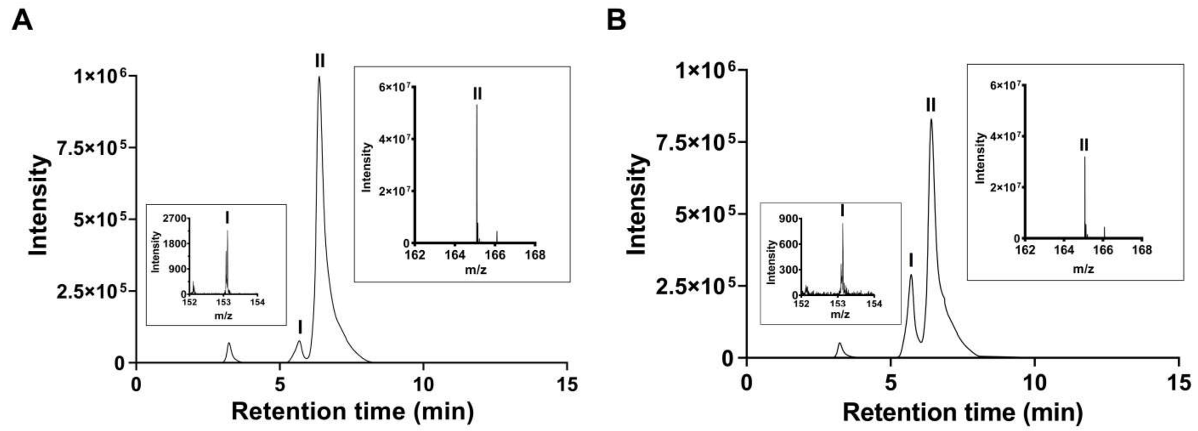

FIG S8 HPLC identification of protocatechuic acid (PCA, peak I) during metabolism (resting cell incubation) of phthalic acid (PA, peak II) by BBP-grown cells of strain PAE-6 in the absence (A) and presence of 1.0 μM $AgNO_3$ (B) as the inhibitor of protocatechuate 3,4-dioxygenase. Insets depict DI-ESI-MS of peak I and peak II.

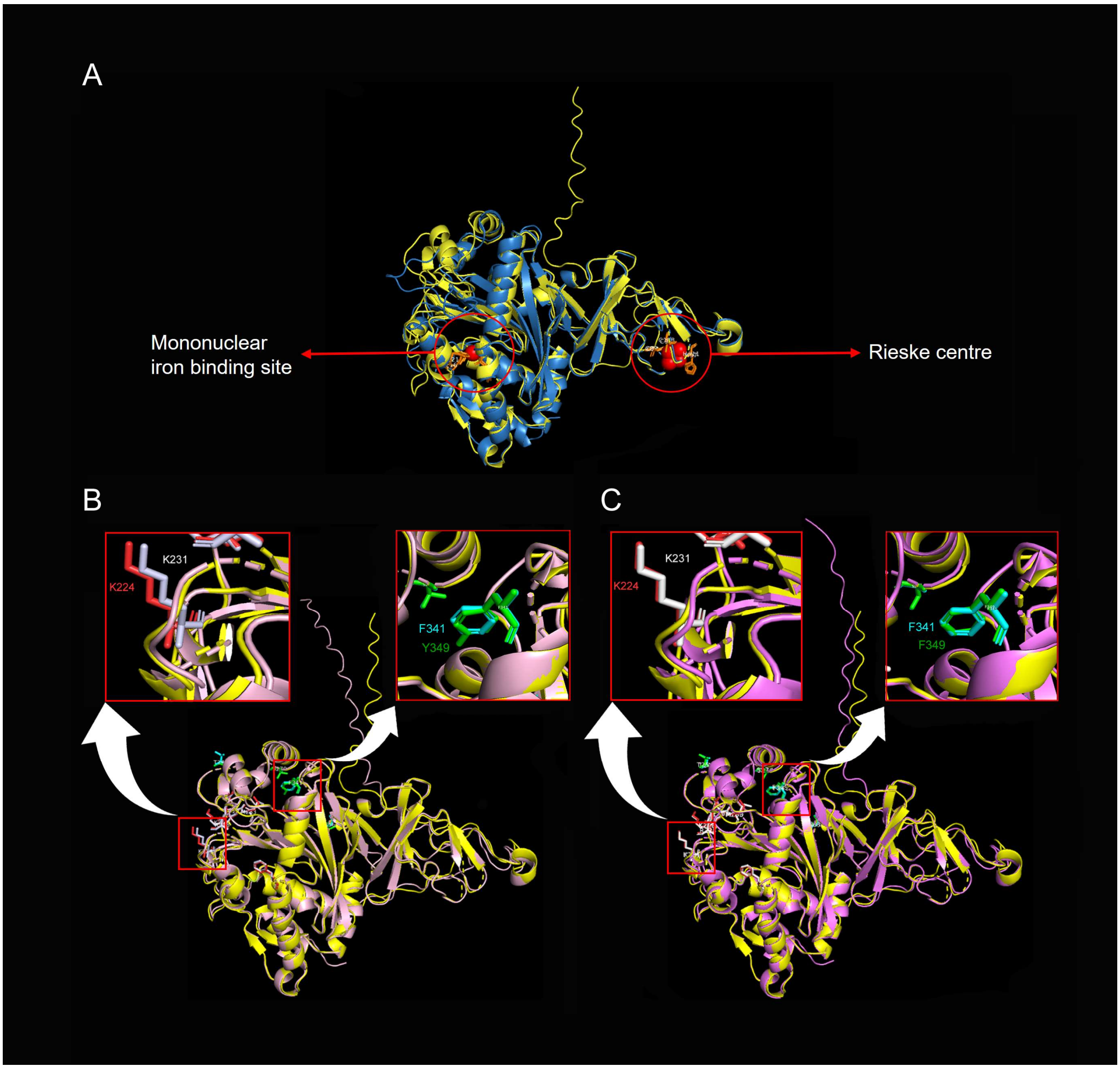

FIG S9 Comparative structural prediction by AlphaFold analysis. (A) A comparison of the alpha subunit of naphthalene dioxygenase (PDB ID: 2B1X) (blue) of *Rhodococcus* sp. strain NCIMB 12038 with the alpha subunit (PadAa$_{RHA1}$) of biochemically well-characterized phthalate 3,4-dioxygenase (yellow) of *Rhodococcus jostii* RHA1 showing aligned mononuclear iron binding site and rieske centre. The interacting amino acid residues are shown in orange. (B) Structural alignment of PadAa$_{RHA1}$ (yellow) and PhtAa$_{PAE-6}$ (light pink) of strain PAE-6. The substrate

binding residues are marked in red and gray for $PadAa_{RHA1}$ and $PhtAa_{PAE-6}$, respectively. The conserved amino acid residues are marked in blue for $PadAa_{RHA1}$ while the altered amino acid residues (Fig. 9) are marked in green Pht forAa_{PAE-6}. Major structural differences are shown in the inset. The RMSD value is 0.456Å. (C) Structural alignment of $PadAa_{RHA1}$ (yellow) and $PhtAa_{PAE-6mut}$ (purple), an *in silico* mutant where the mismatched amino acid residues were restored. The substrate binding residues are marked in red and white for $PadAa_{RHA1}$ and $PhtAa_{PAE-6mut}$, respectively while the conserved amino acid residues are marked in blue for $PadAa_{RHA1}$ and the restored amino acid residues (altered residues that were changed to conserved residues) are marked in green for $PhtAa_{PAE-6mut}$. A major restoration of structural differences (as shown in B) is depicted in the inset. The RMSD value is 0.395Å.

REFERENCES

1. Han C, Tian Y, Zhao J, Yu Z, Jiang S, Guo X, Xiang W, Wang X. 2018. *Microbispora triticiradicis* sp. nov., a novel actinomycete isolated from the root of wheat (*Triticum aestivum* L.). Int J Syst Evol Microbiol 68:3600-3605.
2. Álvarez-Narváez S, Giguère S, Cohen N, Slovis N, Vázquez-Boland JA. 2021. Spread of multidrug-resistant *Rhodococcus equi*, United States. Emerg Infect Dis 27:529.
3. Zhang XY, Fan X, Qiu YJ, Li CY, Xing S, Zheng YT, Xu JH. 2014. Newly identified thermostable esterase from *Sulfobacillus acidophilus*: Properties and performance in phthalate ester degradation. Appl Environ Microbiol 80:6870-6878.
4. Wu J, Liao X, Yu F, Wei Z, Yang L. 2013. Cloning of a dibutyl phthalate hydrolase gene from *Acinetobacter* sp. strain M673 and functional analysis of its expression product in *Escherichia coli*. Appl Microbiol Biotechnol 97:2483-2491.
5. Wang J, Hlaing TS, Nwe MT, Aung MM, Ren C, Wu W, Yan Y. 2021. Primary biodegradation and mineralization of aryl organophosphate flame retardants by *Rhodococcus*-*Sphingopyxis* consortium. J Hazard Mater 412:125238.
6. Pathak A, Green SJ, Ogram A, Chauhan A. 2013. Draft genome sequence of *Rhodococcus opacus* strain M213 shows a diverse catabolic potential. Genome Announc 1:e00144-12.
7. Holder JW, Ulrich JC, DeBono AC, Godfrey PA, Desjardins CA, Zucker J, Zeng Q, Leach AL, Ghiviriga I et al. 2011. Comparative and functional genomics of *Rhodococcus opacus* PD630 for biofuels development. PLOS Genet 7:e1002219.

8. Sabir DK, Grosjean N, Rylott EL, Bruce NC. 2017. Investigating differences in the ability of XplA/B-containing bacteria to degrade the explosive hexahydro-1,3,5-trinitro-1,3,5-triazine RDX. FEMS Microbiol Lett 364:14.
9. Liu YF, Chen J, Liu ZL, Shou LB, Lin DD, Zhou L, Yang SZ, Liu JF, Li W et al. 2020. Anaerobic degradation of paraffins by thermophilic Actinobacteria under methanogenic conditions. Environ Sci Technol 54:10610-10620.
10. Powell JA, Archer JA. 1998. Molecular characterisation of a *Rhodococcus* ohp operon. Antonie Leeuwenhoek 74:175-188.
11. Ollis DL, Cheah E, Cygler M, Dijkstra B, Frolow F, Franken SM, Harel M, Remington SJ, Silman I et al. 1992. The α/β hydrolase fold. Protein Eng 5:197-211.
12. Inoue D, Tsunoda T, Yamamoto N, Ike M, Sei K. 2018. 1,4-dioxane degradation characteristics of *Rhodococcus aetherivorans* JCM 14343. Biodegradation 29:301-310.
13. Crombie AT, Larke-Mejia NL, Emery H, Dawson R, Pratscher J, Murphy GP, McGenity TJ, Murrell JC, Murrell JC. 2018. Poplar phyllosphere harbors disparate isoprene-degrading bacteria. Proc Natl Acad Sci USA 115:13081-13086.
14. van Straaten KE, Gonzalez CF, Valladares RB, Xu X, Savchenko AV, Sanders DA. 2009. The structure of a putative S-formylglutathione hydrolase from *Agrobacterium tumefaciens*. Protein Sci 18:2196-2202.
15. Ren C, Wang Y, Tian L, Chen M, Sun J, Li L. 2018. Genetic bioaugmentation of activated sludge with dioxin-catabolic plasmids harbored by *Rhodococcus* sp. strain p52. Environ Sci Technol 52:5339-5348.
16. Huber L, Giguère S, Slovis NM, Álvarez-Narváez S, Hart KA, Greiter M, Morris ERA, Cohen ND, Cohen ND. 2020. The novel and transferable erm 51 gene confers macrolides, lincosamides and streptogramins B MLSB resistance to clonal *Rhodococcus equi* in the environment. Environ Microbiol 22:2858-2869.
17. Navas LE, Dexter G, Liu J, Levy-Booth D, Cho M, Jang SK, Mansfield SD, Renneckar S, Mohn WW et al. 2021. Bacterial transformation of aromatic monomers in softwood black liquor. Front Microbiol 12:735000.
18. Goins CM, Dajnowicz S, Smith MD, Parks JM, Ronning DR. 2018. Mycolyltransferase from *Mycobacterium tuberculosis* in covalent complex with tetrahydrolipstatin provides insights into antigen 85 catalysis. J Biol Chem 293:3651-3662.
19. Hsiao TH, Chen YL, Meng M, Chuang MR, Horinouchi M, Hayashi T, Wang PH, Chiang YR, Chiang YR. 2021. Mechanistic and phylogenetic insights into Actinobacteria-mediated oestrogen biodegradation in urban estuarine sediments. Microb Biotechnol 14:1212-1227.
20. Garrido-Sanz D, Sansegundo-Lobato P, Redondo-Nieto M, Suman J, Cajthaml T, Blanco-Romero E, Martin M, Uhlik O, Rivilla R, Uhlik O, Rivilla R. 2020. Analysis of the biodegradative and adaptive potential of the novel polychlorinated biphenyl degrader *Rhodococcus* sp. WAY2 revealed by its complete genome sequence. Microb Genomics 6.
21. Navazas A, Thijs S, Feito I, Vangronsveld J, Peláez AI, Cuypers A, González A. 2021. Arsenate-reducing bacteria affect as accumulation and tolerance in Salix atrocinerea. Sci Total Environ 769:144648.

22. Spiller B, Gershenson A, Arnold FH, Stevens RC. 1999. A structural view of evolutionary divergence. Proc Natl Acad Sci USA *96*:12305-12310.

23. Hu R, Zhao H, Xu X, Wang Z, Yu K, Shu L, Yan Q, Wu B, Mo C et al. 2021. Bacteria-driven phthalic acid ester biodegradation: Current status and emerging opportunities. Environ Int 154:106560.

24. Nishioka T, Iwata M, Imaoka T, Mutoh M, Egashira Y, Nishiyama T, Shin T, Fujii T, Fujii T. 2006. A mono-2-ethylhexyl phthalate hydrolase from a *Gordonia* sp. that is able to dissimilate di-2-ethylhexyl phthalate. Appl Environ Microbiol 72:2394-2399.

25. Zhang H, Lin Z, Liu B, Wang G, Weng L, Zhou J, Hu H, He H, Huang Y et al. 2020. Bioremediation of di-2-ethylhexyl phthalate contaminated red soil by *Gordonia terrae* RL-JC02: Characterization, metabolic pathway and kinetics. Sci Total Environ 733:139138.

26. Stanislauskienė R, Rudenkov M, Karvelis L, Gasparavičiūtė R, Meškienė R, Časaitė V, Meškys R. 2011. Analysis of phthalate degradation operon from *Arthrobacter* sp. 68b. Biologija 57.

27. Li D, Yan J, Wang L, Zhang Y, Liu D, Geng H, Xiong L. 2016. Characterization of the phthalate acid catabolic gene cluster in phthalate acid esters transforming bacterium-*Gordonia* sp. strain HS-NH1. Int Biodeterior Biodegrad 106:34-40.

28. Yang T, Ren L, Jia Y, Fan S, Wang J, Wang J, Nahurira R, Wang H, Yan Y, Wang H, Yan Y. 2018. Biodegradation of di-2-ethylhexyl phthalate by *Rhodococcus ruber* YC-YT1 in contaminated water and soil. Int J Environ Res Public Health 15:964.

29. Mande SS, Sarfaty S, Allen MD, Perham RN, Hol WG. 1996. Protein–protein interactions in the pyruvate dehydrogenase multienzyme complex: Dihydrolipoamide dehydrogenase complexed with the bindingdomain of dihydrolipoamide acetyltransferase.Structure 4:277–286.

30. Habe H, Miyakoshi M, Chung J, Kasuga K, Yoshida T, Nojiri H, Omori T. 2003. Phthalate catabolic gene cluster is linked to the angular dioxygenase gene in *Terrabacter* sp. strain DBF63. Appl Microbiol Biotechnol 61:44-54.

31. Dueholm MS, Albertsen M, D'Imperio S, Tale VP, Lewis D, Nielsen PH, Nielsen JL. 2014. Complete genome of *Rhodococcus pyridinivorans* SB3094, a methyl-ethyl-ketone-degrading bacterium used for bioaugmentation. Genome Announc 2:e00525-14.

32. Ren L, Wang G, Huang Y, Guo J, Li C, Jia Y, Chen S, Zhou JL, Hu H, Zhou JL, Hu H. 2021. Phthalic acid esters degradation by a novel marine bacterial strain *Mycolicibacterium phocaicum* RL-HY01: Characterization, metabolic pathway and bioaugmentation. Sci Total Environ 791:148303.

33. Haddad S, Eby DM, Neidle EL. 2001. Cloning and expression of the benzoate dioxygenase genes from *Rhodococcus* sp. strain 19070. Appl Environ Microbiol 67:2507-2514.

34. Frederick J, Hennessy F, Horn U, De la Torre Cortes P, Van Den Broek M, Strych U, Brady D, et al. 2020. The complete genome sequence of the nitrile biocatalyst *Rhodococcus rhodochrous* ATCC BAA-870. BMC Genomics 21:1-19.

35. Gröning JA, Eulberg D, Tischler D, Kaschabek SR, Schlömann M. 2014. Gene redundancy of two-component chloro phenol hydroxylases in *Rhodococcus opacus* 1CP. FEMS Microbiol Lett 361:68-75.
